# Effectiveness and Safety of Interventions for Sarcopenia in Advanced Prostate Carcinoma: Systematic Review

**DOI:** 10.1002/jcsm.70290

**Published:** 2026-05-05

**Authors:** Pedro de Pablos‐Rodríguez, Aythami de Armas‐Castellano, Diego Infante‐Ventura, Tasmania del Pino‐Sedeño, Antonio Rueda Domínguez, Pablo B. Pedrianes‐Martín, Jose A. L. Calbet, Pedro de Pablos‐Velasco, María M. Trujillo‐Martín

**Affiliations:** ^1^ Department of Urology Instituto Valenciano de Oncología (IVO) Valencia Spain; ^2^ Canry Islands Health Research Institute Foundationa (FIISC) Las Palms de Gran Canaria Spain; ^3^ Canary Islands Health Research Institute Foundation (FIISC) El Rosario Santa Cruz de Tenerife Spain; ^4^ Evaluation Unit of the Canary Islands Health Service (SESCS) El Rosario Santa Cruz de Tenerife Spain; ^5^ Network for Research on Chronicity, Primary Care, and Health Promotion (RICAPPS) Madrid Spain; ^6^ Medical Oncology Intercenter Unit Regional and Virgen de la Victoria University Hospitals, IBIMA Málaga Spain; ^7^ Department of Endocrinology and Nutrition University Hospital of Gran Canaria Doctor Negrín Las Palmas de Gran Canaria Spain; ^8^ Research Institute of Biomedical and Health Sciences (IUIBS) University of Las Palmas de Gran Canaria (ULPGC) Las Palmas de Gran Canaria Spain; ^9^ Department of Physical Education University of Las Palmas de Gran Canaria Las Palmas de Gran Canaria Spain

**Keywords:** meta‐analysis, muscle, prostatic neoplasm, quality of life, sarcopenia, systematic review

## Abstract

**Background:**

Sarcopenia has emerged as a potential prognostic factor in patients with advanced prostate cancer (PCa), requiring interventions for its prevention and treatment.

**Objective:**

We aimed to systematically identify, critically assess and synthesize the available evidence on the effectiveness and safety of interventions for preventing or treating sarcopenia in advanced PCa patients.

**Methods:**

MEDLINE, Embase and Web of Science were searched. Randomized and non‐randomized controlled trials or longitudinal observational studies with a control group focusing on PCa patients aged 60 years and older were considered. Study selection, data extraction and risk‐of‐bias assessment of the included studies were performed in duplicate. When possible, pooled effect estimates were calculated.

**Results:**

Twenty studies (*n* = 1275) were included. Resistance training (RT) (MD = 3.22 kg; 95% CI 0.69, 5.75) and the use of antimyostatin peptibody (MD = 2.2 kg; SE 0.8%) demonstrated statistically significant prevention of lean body mass loss in men undergoing androgen deprivation therapy (ADT). Exercise improved leg press (MD = 25.17 kg; 95% CI [8.71, 41.62]), leg extension (MD = 9.63 kg; 95% CI [4.83, 14.42]), seated row (MD = 4.38 kg; 95% CI [1.54, 7.22]) and chest press strength (MD = 1.70 kg; 95% CI [−1.48, 4.88]) and enhanced patients' physical functioning in chair sit‐to‐stand tests (MD = −1.02 kg; 95% CI [−1.70, −0.34]). RT improved health‐related quality of life (HRQoL) in both general and specific domains and also reduced somatization (MD = −0.69 kg; 95% CI [−1.32, −0.07]) and psychological distress (MD = −1.63 kg; 95% CI [−3.10, −0.15]).

**Conclusions:**

The findings highlight the potential benefits of RT and selected pharmacological interventions on muscle‐related and functional outcomes. However, the significant heterogeneity and lack of comprehensive outcome reporting underscore the need for more standardized and long‐term research through larger, well‐designed randomized controlled trials with standardized measurement methods to draw conclusive evidence and enhance the reliability and applicability of findings in clinical practice.

## 1 Introduction

Prostate cancer (PCa) represents a pressing global health challenge, with an estimated 1.4 million cases diagnosed worldwide each year. It is the second most common type of cancer in men, after lung cancer, and ranks fifth among cancer‐related deaths [1]. The mortality rate of PCa has shown a decreasing trend since the mid‐1990s, largely attributed to advances in treatment options and enhanced detection rates through more rigorous screening protocols [2, 3].

Despite significant advances in medical therapy based on our better understanding of PCa cell biology, androgen deprivation therapy (ADT) remains the cornerstone component of treatment for PCa [4].

The primary objective of ADT is to suppress androgen stimulation in the cancer cells, which, in advanced PCa, leads to a delay in the progression of the disease. However, ADT can have significant adverse effects that negatively impact the patient's quality of life. These may include sexual dysfunction, metabolic and bone alterations and cardiovascular risks [5]. Metabolic and bone alterations resulting from ADT can lead to bone demineralization, increased abdominal fat and skeletal muscle atrophy [5, 6].

The progressive loss of skeletal muscle mass and function, known as sarcopenia, is typically associated with ageing. Sarcopenia affects approximately 6%–22% of older adults, constituting a widespread degenerative condition directly linked to falls, fractures, physical frailty, fatigue, hospitalization and mortality [7]. Additionally, though it has a weak association with shorter overall survival (OS), sarcopenia is an important prognostic factor for progression‐free survival (PFS) in patients with advanced PCa [8]. Moreover, this condition places a significant economic burden on the healthcare system. In 2014, the estimated annual cost of hospitalization for individuals with sarcopenia in the United States was US $40.4 billion, with an average per‐person cost of US $260 [9]. In the second meeting of the European Working Group on Sarcopenia in Older People (EWGSOP), three indicators for diagnosing sarcopenia were identified: muscle strength, muscle quality or quantity and physical performance [10]. In this regard, sarcopenia differs from cancer cachexia in that it is not specifically associated with weight loss and is not necessarily characterized by intense inflammatory processes. Cancer cachexia implies a reduction in fat‐free mass, but also in fat mass that has been linked to decreased health‐related quality of life (HRQoL) and OS [11]. Nevertheless, these two syndromes overlap, especially in older patients, where sarcopenia can be considered as a component of cachexia, but not the other way around [12].

Interventions during and after ADT are crucial for preventing the development of sarcopenia and for attenuating the deleterious effects of ADT in skeletal muscle and bone metabolism and consequently in muscle strength and physical function [6]. Given the typically slow clinical evolution of PCa, interventions targeting muscle mass, strength and physical performance are considered clinically relevant and can be implemented throughout the treatment to mitigate its consequences.

Physical exercise programmes or multicomponent programmes that include physical exercise are considered potential counter‐measures against sarcopenia and the adverse effects of ADT.

These programmes have been shown to have beneficial impacts on muscle mass, muscle strength and physical performance in both healthy and sarcopenic older adults [13, 14]. Furthermore, exercise‐based interventions have proven to be valuable in preserving muscle mass and functionality in critically ill patients in high‐dependency units or intensive care units [15]. PCa survivors have experienced attenuation of treatment side effects and improvements in their quality of life with significant results with interventions including exercise [16, 17, 18]. Moreover, nutritional supplementation as a complement to physical exercise has also demonstrated promising effects [19, 20].

However, it is important to note that the outcomes of these programmes may vary due to treatment‐related acute changes in physical performance and well‐being. In some cases, they may offer palliative rather than recuperative effects [21]. Therefore, further investigation is needed to assess the benefits of such programmes and other interventions in specific populations, such as patients with advanced PCa.

The aims of this review were to identify, critically assess and synthesize the available scientific evidence on the effectiveness and safety of interventions for the prevention or treatment of sarcopenia in patients with advanced PCa.

## 2 Evidence Acquisition

A systematic review was conducted following the Cochrane Handbook for Systematic Reviews of Interventions methodology [22] and is reported in accordance with the Preferred Reporting Items for Systematic Reviews and Meta‐Analyses (PRISMA) guidelines [23]. The review protocol was registered in the PROSPERO database (reference number CRD42021248651).

### 2.1 Information Sources and Search Strategy

We searched MEDLINE (using the Ovid platform), Embase and Web of Science (WOS) databases (from database inception to 20 January 2023). The search strategy was initially developed in MEDLINE, including both controlled vocabulary and text‐word terms, and was subsequently adapted for the other databases. The search combined terms related to sarcopenia and prostate neoplasms. Searches were limited to the English and Spanish languages. The detailed search strategies can be found in Table S1. Additionally, the reference lists of all relevant papers were scrutinized to identify additional studies meeting the selection criteria.

### 2.2 Study Selection Criteria

Studies were considered eligible for inclusion if they met the following criteria:

- Study design: randomized controlled trials (RCTs), non‐RCTs (nRCTs) or longitudinal observational studies (LOSs) with a control group were included. Excluded designs were LOSs without a control group, cross‐sectional studies, case series or case studies and systematic or narrative reviews.
- Population: studies involving men aged 60 years and older diagnosed with PCa across the following cancer stages were included: (1) localized cancer, defined as Stage T1–T2 tumour but with N1 or NX lymph node involvement and/or with M1 or MX metastases; (2) locally advanced cancer, defined as Stage T3–T4 tumour with or without N0 or N1 lymph node involvement and with or without M0 or M1 metastases; (3) cancer with nodal or non‐nodal metastasis, defined as Stage T3–T4 N0 M0 Tx N1 with lymph node metastasis or M1 metastasis.
  Studies were also considered if they included a subset of the participants relevant to the review question, such as patients under 60 years old or those with other types of cancer in addition to patients with PCa, provided that the results for patients meeting the inclusion criteria were reported separately or they represented more than 80% of the target population. Given the importance of ADT in PCa treatment, especially in cases of high‐risk localized, locally‐advanced non‐metastatic and metastatic disease [4], studies focusing on patients with PCa undergoing ADT were incorporated.
- Intervention: any intervention intended to prevent or treat sarcopenia, including but not limited to nutritional or exercise programmes, drug treatments and their combination.
- Comparator: usual care, no intervention, sham intervention or placebo.
- Outcome measures: studies reporting on sarcopenia, defined as a progressive and generalized loss of skeletal muscle mass, area or volume (or skeletal muscle index‐SMI), along with function, assessed by magnetic resonance imaging (MRI), computed tomography scan (CTs), dual‐energy X‐ray absorptiometry (DXA) or bioelectrical impedance analysis (BIA) were included. Additionally, studies reporting OS, cancer‐specific survival (CSS), muscle mass, muscle strength, physical function, adverse events, complications of cancer or HRQoL and other patient‐reported outcomes measures (PROMs) were considered.

### 2.3 Study Selection Process

Two reviewers separately and independently screened all retrieved citations, initially evaluating the titles and abstracts. Subsequently, the full‐text articles that appeared to fulfil the pre‐established selection criteria were assessed. Any disagreements or uncertainties were resolved through discussion between the reviewers, with the involvement of a third one if necessary.

### 2.4 Data Collection Process

An Excel‐based data extraction form was created, pilot‐tested on two studies and subsequently refined based on feedback. Two reviewers, blinded to each other's assessments, extracted data from the selected studies. This included details such as article identification (author, publication date, country and funding), study design and methodology (context and duration), population characteristics (selection criteria and demographics), interventions (type, modality and number of sessions), comparators, outcomes (definition, measurement method and value) and results.

### 2.5 Risk of Bias Assessment

The risk of bias in the included studies was assessed using the Cochrane Collaboration tools: Risk of Bias 2 (RoB 2) [24] for RCTs and ROBINS‐I [25] for LOS with control group. Two reviewers independently assessed the risk of bias, resolving discrepancies through discussion. If consensus could not be reached, a third reviewer was consulted.

### 2.6 Effect Measures

The extent of the post‐treatment effects of the interventions was evaluated in terms of OS and CSS. Hazard ratio (HR) and its 95% confidence intervals (95% CI) were estimated. The effect on muscle mass, physical function, HRQoL and other PROM were estimated as mean differences (MD) or standardized MD (SMD), with their corresponding 95% CI.

### 2.7 Analysis and Synthesis of Results

The information collected from the results of each included study was synthesized narratively. A quantitative synthesis of the results was conducted through meta‐analysis for outcomes reported by two or more studies. Post‐treatment MD or SMD were estimated using the inverse variance method [26, 27] using the *metagen* and the *forest.meta* functions of the meta package for R version 4.3.0. When single studies did not report between‐group post‐intervention MDs and 95% CI, they were calculated using Review Manager (RevMan, version 5.4.1. Copenhagen: the Nordic Cochrane Center, the Cochrane Collaboration, 2020).

HR and the corresponding 95% CI were pooled with an indirect variance estimation in meta‐analyses using RevMan.

Heterogeneity was assessed graphically by presenting the estimated effects and their 95% CI of each study in a forest plot, as well as by the Higgins' *I* ^2^ statistic [28]. When there was heterogeneity (*I* ^2^ ≥ 50% or *p* < 0.1), meta‐analyses were performed using the restricted maximum likelihood (REML) random effects model. When an outcome measure was examined by more than one test by one study, the study sample was divided equally among the selected tests.

A sensitivity analysis was conducted by omitting each study individually to determine the stability of the overall estimate of the effect.

The effects of intervention type and duration of intervention were explored using subgroup analyses or meta‐regression when the number of studies was considered sufficient (*k* ≥ 10). The nature of the data reported for the pre‐specified grouping variables of age, presence of metastases, disease stage and sarcopenia stage did not allow studies to be grouped for the analysis following these variables.

### 2.8 Publication Bias Assessment

Whenever the number of studies and available data allowed, publication bias was assessed by visual inspection of funnel plots and by computing the Egger regression test. If 10 or more studies were included in a meta‐analysis, the significance level was set to 0.05. These analyses were performed using the *funnel.meta* and the *metabias* functions from the meta package for R 4.3.0, respectively.

### 2.9 Certainty of Evidence Assessment

The certainty of evidence (CoE) for each outcome was assessed using the Grading of Recommendations Assessment, Development and Evaluation (GRADE) approach. Certainty was rated down considering the risk of bias, inconsistency, indirectness, imprecision and publication bias. Conversely, it was rated up considering large effect size, dose–response gradient and plausible confounding that would have otherwise weakened the effect estimate [29]. Evidence profiles were created, and the overall CoE was rated from very low (indicating little confidence in the estimate, with the true effect likely to differ substantially from the estimate) to high (reflecting a high level of confidence that the true effect is close to that of the estimate). All CoE assessments including RCTs can be found as Data S1.

## 3 Evidence Synthesis

**FIGURE 1:**
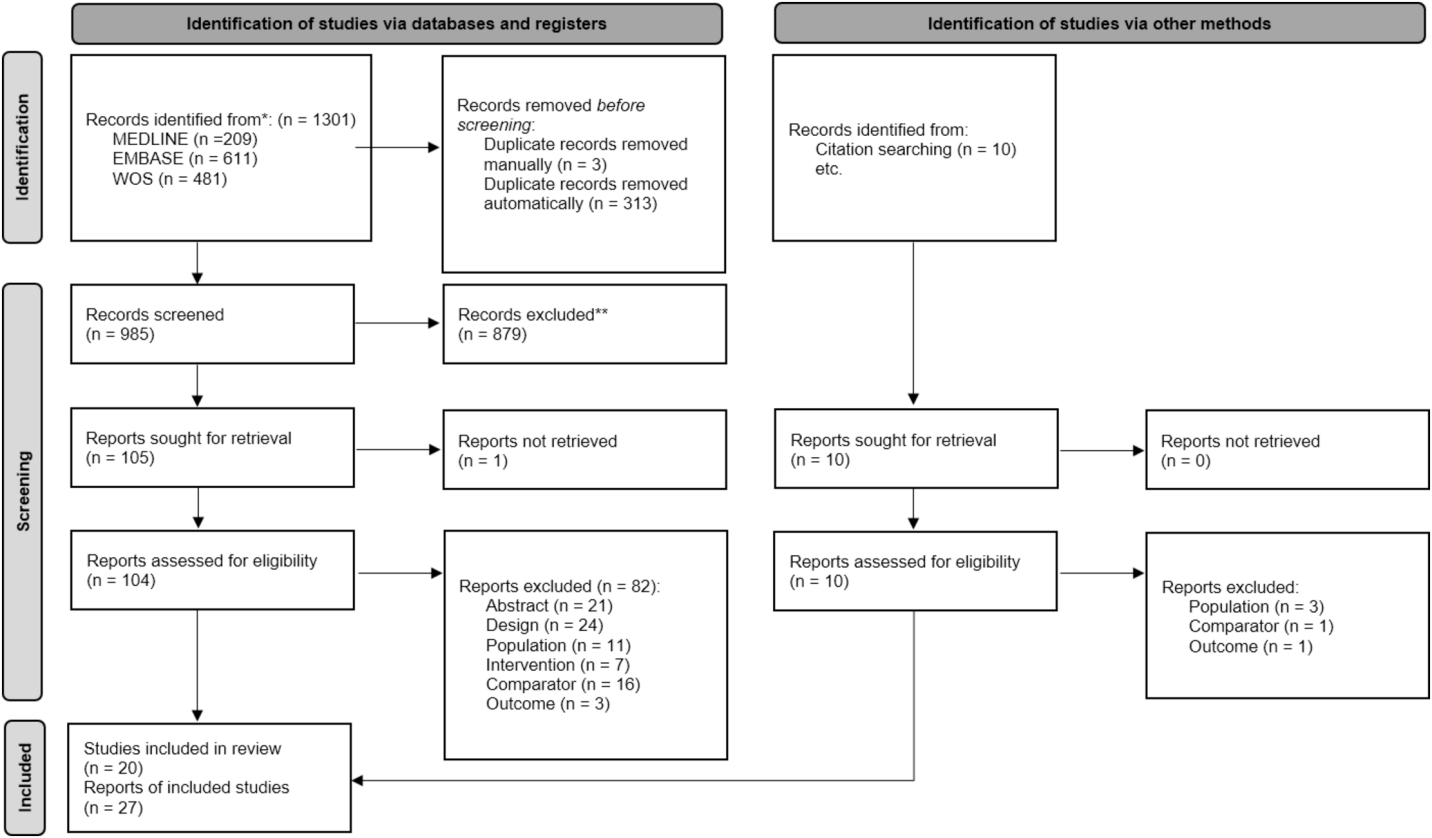
PRISMA flow diagram.

The results of the literature search and study selection process are shown in Figure 1. The search identified 995 manuscripts of which 115 potentially relevant articles were selected for full‐text assessment. Twenty original studies [30, 31, 32, 33, 34, 35, 36, 37, 38, 39, 40, 41, 42, 43, 44, 45, 46, 47, 48, 49] reported in 27 papers [30, 31, 32, 33, 34, 35, 36, 37, 38, 39, 40, 41, 42, 43, 44, 45, 46, 47, 48, 49, 50, 51, 52, 53, 54, 55, 56] met the pre‐established criteria and were included. All selected studies were published in English between 2003 and 2023. The list of studies excluded during the full‐text assessment is available in Table S2.

### 3.1 Description of Included Studies

The main characteristics of the included studies are summarized in Table 1. Eighteen studies were RCTs [30, 31, 32, 33, 34, 36, 37, 38, 39, 40, 41, 42, 43, 45, 46, 47, 48] while two were LOS with control group [35, 44], one retrospective [35] and one prospective [44]. Most studies had two arms [30, 31, 32, 34, 37, 38, 40, 42, 43, 44, 46, 47, 48, 49] comparing one intervention with usual care or placebo. Four studies had three arms [35, 36, 41, 45], and one study, which tested different doses of antimyostatin peptibody (AMG745), had four arms [39].

**TABLE 1:** Characteristics of included studies.

Study, year (country)	Study design	*N*	*N* per arm (arms)	Mean age ± SD (range)	Inclusion criteria	Exclusion criteria	Definition of advanced PCa	Interventions	Control	Outcomes
Bourke, 2014 [30]^a^ Bourke, 2011 [50] Gilbert, 2016 [52] (UK)	RCT	100	50/50 (2)	71 ± 7.04 (53–87)	Histologically confirmed non‐localized PCa. Receiving ADT for at least 6 months. Sedentary.	Unstable angina, uncontrolled hypertension, recent myocardial infarction, pacemakers. Painful or unstable bone metastases. Regular physical activity.	PCa patients receiving ADT for at least 6 months	Lifestyle intervention (ExTP + dietary advice)	Usual care	HRQoL, fatigue, aerobic exercise tolerance (treadmill walk time), skeletal muscle mass
Cormie, 2013 [31] (Australia)	RCT	20	10/10 (2)	72.15 ± 7.1 (NR)	Aged 57–83 years. PCa histological diagnosis. Bone metastatic disease.	Moderate–severe bone pain that limit activities of daily living. Musculoskeletal, cardiovascular and/or neurological disorders that could inhibit patients from exercising.	Patients that previously received ADT; bone metastatic disease secondary to PCa	ExTP (resistance)	Usual care	Lean body mass, knee extension strength, fatigue, HRQoL, psychological distress
Cormie, 2015 [32] (Australia)	RCT	63	32/31 (2)	68.82 ± 7.04 (NR)	PCa histological diagnosis. Treatment with leuprorelin acetate depot. Remaining on ADT for at least the next 3 months.	Previously received ADT. Bone metastatic disease. Unable to walk 400 m unassisted or musculoskeletal, cardiovascular and/or neurological disorders that could inhibit from exercising.	PCa patients receiving ADT	ExTP (resistance and aerobic)	Usual care	Whole body lean mass, appendicular lean mass, cardiovascular fitness, lower and upper body max. Strength, lower body function, dynamic balance, static balance, falls self‐efficacy
Galvão, 2010 [33]^a^ Buffart, 2014 [51] (Australia)	RCT	57	29/28 (2)	69.8 ± 7.24 (NR)	Prior exposure to ADT longer than 2 months. Without PSA evidence of disease activity. Hypogonadal for the subsequent 6 months.	Bone metastatic disease. Unable to walk 400 m unassisted or musculoskeletal, cardiovascular and/or neurological disorders that could inhibit from exercising. Resistance training in the previous 3 months.	PCa patients receiving ADT for at least 2 months	ExTP (resistance and aerobic)	Usual care	Whole body lean mass, regional lean mass, dynamic muscle strength, muscle endurance, functional performance, balance, falls self‐efficacy, quality of life status and cancer specific QoL
Focht, 2018 [48] (USA)	RCT	32	16/16 (2)	NR ± NR (NR)	Histologically defined diagnosis of prostate cancer based on pathology reports and staging studies. Currently undergoing androgen‐deprivation therapy with a planned course of at least 3 months of continuous therapy. Sedentary activity pattern with less than 60 min of structured exercise participation per week during the past 6 months. Free of poorly controlled medical conditions that precluded safe participation in an exercise programme. Consent to participate from the treating oncologist and primary care physician. Willingness to accept randomization and undergo the testing and intervention procedures.	NR	PCa patients receiving ADT for at least 3 months	Lifestyle intervention (ExTP + plant‐based dietary advice + CBI)		Fat‐free mass, leg extension strength, chest press strength, 400 m walk time, stair climb time, adverse events.
Galvão, 2018 [34] (Australia)	RCT	57	28/29 (2)	70 ± 8.4 (NR)	Bone metastases No acute illness No significant bone pain as assessed by their clinician.	Musculoskeletal, cardiovascular or neurological disorders that could inhibit patients from exercising. Structured and supervised aerobic and/or resistance training.	Bone metastasis	ExTP (M3EP)	Usual care	Lean body mass, self‐reported physical function, knee extension strength, usual 6‐m walk, fast 6‐m walk, 400‐m walk, up and go, sensory organization test
Hojan, 2016 [46] (Poland)	RCT	55	27/28 (2)	68.5 ± 6.14 (NR)	Age between 18 and 75 years. Scheduled ADT planned to continue for a total period of 36 months. A total RT dose of 76 Gy in 38 fractions. ECOG PS of 0–1	Distant metastases and/or disease progression resulting in RT or the introduction of chemotherapy. Insufficiently controlled arterial hypertension or cardiac disease resulting in circulation failure. Insufficiently controlled metabolic disease. Endocrinological, rheumatic and absorption disorders. Tumours outside of the prostate.	PCa patients receiving ADT for at least 3 months	ExTP (resistance and aerobic)	Usual care	Six‐minute walk test distance, HRQoL, cancer specific QoL, fatigue.
Hong, 2013 [35] (South Korea)	ROS	109	20/21/68 (3)	68.4 ± 7.4 (45–87)	PCa histological diagnosis. Progressive visceral metastases or progressive regional lymph node disease after ADT.	< 3 docetaxel cycles. ECOG performance status > 3.	Castration‐resistant PCa; Gleason score ≥ 7	High‐dose and low‐dose megestrol acetate	No megesterol acetate	Overall survival, disease‐specific survival
Houben, 2023 [36] (The Netherlands)	RCT^b^	96	30/30/36 (3)	71.62 ± 7 (NR)	1. Treatment with ADT for at least 6 months.	Comorbidities severely compromising physical activity. High risk for pathological fractures due to bone metastases. Life expectancy < 1 year. Lactose intolerant or whey protein allergy. Cognitive disorders or severe emotional instability. Unable to speak, understand or read the Dutch language.	PCa patients receiving ADT for at least 6 months	ExTP + protein and ExTP + placebo	Usual care	Lean body mass, skeletal muscle mass, max. Strength, physical performance
Lam, 2019 [37] (Australia)	RCT	24	13/11 (2)	70.4 ± 1.5 (NR)	Aged 50–80 years. Histologically confirmed PCa of early or locally advanced stage or metastatic disease with bone involvement only (≤ 5 sites of metastases). Scheduled to receive conventional ADT with gonadotrophin‐releasing hormone analogs. ECOG PS of 0.	Concurrent chemotherapy or previous ADT within the last 12 months. Musculoskeletal, cardiovascular or neurological disorders which prevent participants from undertaking upper and lower limb exercises.	PCa patients receiving ADT; Gleason score ≥ 7	ExTP (resistance)	Usual care	Lean body mass
Ndjavera, 2020 [47] (UK)	RCT	50	24/26 (2)	72 ± 4.8 (NR)	Histologically confirmed prostate cancer. Aged 50–80 years. Beginning LHRH agonist treatment with or without radiotherapy. Anticipated to remain on ADT for ≥ 6 months. Be classified as 0 or 1 according to the WHO performance status. Not achieving 150 min/week of moderate intensity physical activity during the last 6 months.	Metastatic bone disease. Previously treated with ADT. Involvement in any other clinical trial. Prior CV event or heart failure. Chronic obstructive. Pulmonary disease. An absolute contraindication to exercise testing or training.	PCa patients receiving ADT for at least 6 months	ExTP (resistance and aerobic)	Usual care	Fat‐free mass, hand‐grip strength, cancer specific QoL, fatigue, adverse events
Nilsen, 2015 [49] (Norway)	RCT	58	28/30 (2)	NR ± NR (NR)	Referred to high‐dose radiotherapy, starting 2–6 months after the initiation of neo‐adjuvant ADT, followed by adjuvant ADT, which continued for 9–36 months. Age ≤ 75 years. Ability to understand Norwegian Residence less than 1 h by car from the training facility.	Regular strength training (≥ 1 session per week) Use of osteoporosis medication Medical conditions that could complicate participation	PCa patients receiving ADT for between 2 and 6 months	ExTP (resistance)	Usual care	Lean body mass, leg, chest and shoulder press strength, sit‐to‐stands in 30 s, stair climb time
Nilsen, 2016 [38, p. 20] (Norway)	RCT	37	19/18 (2)	NR ± NR (NR)	Intermediate or high‐risk profile.^c^ Aged ≤ 75 years. Ability to understand Norwegian. Residence less than 1 h by car from the training facilities.	Regular strength training. Use of osteoporosis medication. Conditions that could complicate participation.	Intermediate or high‐risk profile^c^	ExTP (resistance)	Usual care	Knee extension strength
Padhi, 2014 [39] (USA)	RCT	54	31/23 (2)	73.3 ± 6.7 (NR)	No documented distant metastasis. Received ADT for at least 6 months as a primary, adjuvant or salvage treatment. If ADT was being administered intermittently, serum total T level less than 50 ng/dL at screening. Stable PSA. No history of primary muscle disease, myopathy, or neuropathy. Weight 137 kg or less. Height 198 cm or less.	NR	PCa patients receiving ADT for at least 6 months	Antimyostatin peptibody (AMG 745)	Placebo	Lean body mass, adverse events, lower extremity muscle size.
Rhee, 2021 [40, p. 20] (Australia)	RCT	50	25/25 (2)	69.5 ± NR (65–74)	Aged 18–80 years. Commencing or receiving ADT.	Non‐prostatic metastatic disease. Coexisting conditions that affect LBM such as liver disease, renal insufficiency and diabetes mellitus. Systemic illness that required medications such as glucocorticoids. Thyroid dysfunction. Participation in an exercise or weight‐control programme. Compromised physical mobility that required assistance with daily living.	PCa patients receiving ADT	Unesterified Crystalline USP grade oral testosterone	Placebo	Lean body mass
Sajid, 2016 [41, p. 201] (USA)	RCT	19	8/6/5 (3)	75.43 ± 7.33 (NR)	Aged ≥ 65 years. ADT for at least 3 months for histologically confirmed PCa. Stable disease on ADT (stable or declining PSA, no progressive clinical symptoms and no new metastatic lesions for 1 month prior to study entry). Sedentary	Possible cognitive impairment, > 5 errors on the Short Portable Mental Status Questionnaire (SPMSQ). Significant physical performance impairment. Active or maintenance physical activity. Patients planning to change their exercise behaviour.	PCa patients receiving ADT for at least 3 months	Home‐based walking and resistance ExTP; technology‐mediated walking and resistance ExTP	Usual care	Lean muscle mass, short physical performance battery (SPPB), handgrip strength, steps per day, chest press reps
Segal, 2003 [42] (Canada)	RCT	155	82/73 (2)	67.96 ± 7.69 (NR)	Histologically documented PCa. ADT for at least 3 months after recruitment.	Severe cardiac disease. Uncontrolled hypertension (blood pressure > 160/95 mmHg). Uncontrolled pain. Unstable bone lesions. Residence more than 1 h from the study center.	PCa patients receiving ADT for at least 3 months; cancer stage ≥ T2	ExTP (resistance)	Waitlist control	Fatigue, HRQoL, muscular fitness, anthropometric measures
Uth, 2014 [43]^a^ Uth, 2016 [53] (Denmark)	RCT	57	29/28 (2)	NR ± NR (NR)	Locally advanced or advanced PCa managed with medical or surgical ADT for at least 6 months. Aged 18–76 years. Ability to read and understand Danish.	WHO performance level > 1. Osteoporosis. VO2 max > 35 mL/kg/min Activity limiting pain from bone metastasis. Cardiovascular or pulmonary disorders. Anticoagulant therapy. Abnormal screening blood samples. Abnormal liver function. Coagulopathy. Malignant disease other than PCa. Current or scheduled chemotherapy.	PCa patients receiving ADT for at least 6 months	Football training programme	Usual care	Lean body mass, knee extension strength, jump height, sit‐to‐stand test, 9‐step staircase climbing, bipedal stance, tandem stance, flamingo balance test
Uth, 2018 [44] (Denmark)	POS	22	11/11 (2)	NR ± NR (NR)	Locally advanced or advanced PCa managed with medical or surgical ADT for at least 6 months. Aged 18–76 years. Ability to read and understand Danish.	WHO performance level > 1. Osteoporosis. Activity limiting pain from bone metastasis. Cardiovascular or pulmonary disorders. Anticoagulant therapy. Abnormal screening blood samples. Abnormal liver function. Coagulopathy. Malignant disease other than PCa. Current or scheduled chemotherapy.	PCa patients receiving ADT for at least 6 months	Football training programme	Usual care	Lean body mass, knee extension strength, sit‐to‐stand test, flamingo balance test
Wall, 2017 [45]^a^ Galvao, 2021 [57] Newton, 2019 [56] Taaffe, 2017 [54] (Australia)	RCT	97	50/47 (2)	69.1 ± 8.88 (43–90)	Histologically documented localized PCa. Hypogonadal for the duration of the study.	Already undergoing structured exercise. Unable to walk 400 m. Bone metastatic disease.	PCa patients receiving ADT for at least 2 months; Gleason score ≥ 7	ExTP (resistance and aerobic)	Usual care	Lean body mass, seated row, chest press, leg press and leg extension strength, sit‐to‐stand test, 400‐m walk, 6 m backwards walk, psychological distress,

Abbreviations: ADT, androgen deprivation therapy; CBI, cognitive‐behavioural intervention; ECOG, Eastern Cooperative Oncology Group; ExTP, exercise training programme; HRQoL, health‐related quality‐of‐life; M3EP, modular multi‐modal physical exercise programme; NR, not reported; PCa, prostate cancer; POS, prospective observational study; PSA, prostate specific antigen; QoL, quality‐of‐life; RCT, randomized controlled trial; ROS, retrospective observational study; UK, United Kingdom; USA, United States of America.

^a^ Considered as main publication, referenced in text.

^b^ Allocation to intervention groups was random, control group was recruited separately.

^c^ Following D'Amico et al. 2003 criteria (28).

The studies were conducted in eight different countries: Australia [31, 32, 33, 34, 37, 40, 45], Denmark [43, 44], the United States [39, 40, 48], the United Kingdom [47, 50], Canada [42], Norway [38, 49], Poland [46], South Korea [35] and the Netherlands [36]. Across the 20 studies, a total of 1275 men were recruited. The largest and the smallest studies included 160 [45] and 19 men [41], respectively. The mean age (SD) of patients from the 16 studies that provided complete information on patient age [30, 31, 32, 33, 35, 36, 37, 39, 41, 42, 45, 46, 47, 48, 49] was 69.6 (7.6) years, with ages ranging from 43 to 90 years.

Regarding the definition of advanced PCa patients, 16 studies considered PCa patients receiving ADT [30, 32, 33, 36, 37, 39, 40, 41, 42, 43, 45, 46, 47, 48, 49] as advanced PCa patients, although some of these studies also included additional criteria such as Gleason score ≥ 7 [37, 45] and cancer stage ≥ T2 [42]. Two studies considered bone metastases as an indicator of advanced PCa [31, 34]; one study considered castration resistant PCa with a Gleason score ≥ 7 [35] and one study considered an intermediate or high‐risk profile [38], following the criteria by D'Amico et al. [58].

Fifteen studies evaluated interventions based on various types of physical activity or exercise programmes [31, 32, 33, 34, 36, 37, 38, 41, 42, 43, 44, 45, 46, 47, 49], including one study that also combined an exercise programme with protein supplementation (ET + PRO) [36]. One study focused on a lifestyle intervention (LSI) [30], which consisted of exercise programmes and dietary advice, whereas another study implemented an LSI that included a group‐mediated cognitive behavioural resistance exercise (GMCB‐RET) and dietary intervention [48]. Three studies assessed pharmacological interventions with megesterol acetate (MA) [35], AMG745 [39] and testosterone [40].

In studies incorporating dietary advice, this component of the intervention consisted of advice aimed at improving overall diet quality [30, 48].

### 3.2 Outcome Measures

None of the included studies assessed sarcopenia as a categorical clinical condition using established criteria. Instead, studies reported on muscle‐related and functional outcomes that represent key domains of sarcopenia. One study reported on CSS [35].

Muscle mass was the most reported outcome among studies, being informed on in 16 of the 20 included studies [30, 31, 32, 33, 34, 36, 37, 39, 40, 41, 43, 44, 45, 47, 48, 49]. More specifically, lean body mass (kg) measured through DXA was reported in 15 studies [31, 32, 33, 34, 36, 38, 39, 40, 42, 43, 44, 46, 47, 48]. Other reported measures of muscle mass included appendicular lean mass [32, 33, 36, 49], skeletal muscle mass [30], lean lower and upper limb mass [33] and lower limb muscle size [39].

Fourteen studies reported on muscle strength, measured by a wide variety of strength tests [31, 32, 33, 34, 36, 38, 39, 41, 43, 44, 45, 47, 48, 49]. Lower body strength was measured using one‐repetition maximum weight (1RM) in kilograms (kg) lifted in leg press [32, 33, 45, 49], leg extension [33, 45, 48] and knee extension [31, 33, 38, 43, 44] and by the percentage increase in 1RM leg press and leg extension [36]. Upper body strength was also measured using 1RM in chest press [32, 33, 41, 45, 48, 49], seated row [32, 33, 45], shoulder press [49] and handgrip strength [41, 47].

Physical functioning was also assessed using a broad range of tests in nine studies [30, 32, 33, 34, 36, 39, 41, 43, 44]. Chair sit‐to‐stand tests were performed to measure physical functioning in eight studies [32, 33, 36, 43, 44, 45, 49, 50]; six studies performed stair‐climb tests [32, 33, 36, 43, 48, 49]; five studies reported on 400‐m walk tests [32, 33, 34, 45, 48]; three studies performed 6‐m walk tests [32, 33, 34]; two studies performed a timed up‐and‐go test [34, 36]; one study conducted 6‐min walk tests [46] and another study performed treadmill walk time to measure exercise tolerance [30]. Only one study reported on jump height [43], and another study reported on steps walked per day and on the Short Physical Performance Battery (SPPB) test [41].

Balance was evaluated in five studies [32, 33, 34, 43, 44] by various tests. Three studies reported on the Sensory Organization Test (SOT) [31, 33, 34]. Three studies performed a 6‐m backwards walk test [32, 33, 45], two studies reported on the Activities‐Specific Balance Confidence Scale (ABC) [32, 33] and another two studies performed flamingo balance tests [43, 44]. Only one study reported on bipedal and tandem stances sway area (mm^2^) [43].

Several PROMs were assessed in the included studies. HRQoL was measured in nine studies [30, 31, 32, 34, 42, 46, 47, 49, 50]. General HRQoL was measured in three studies using the Short Form Health Survey (SF‐36) [31, 32, 34], in two studies using the European Organization for Research and Treatment of Cancer (EORTC) Quality of Life Questionnaire for patients with cancer (QLQ‐C30) [46, 49] and in two studies using the Functional Assessment of Cancer Therapy‐General (FACT‐G) tool [30, 46]. Disease‐specific HRQoL was measured in two studies using the EORTC Quality of Life Questionnaires for patients with PCa (QLQ‐PR25 and QLQ‐PR23) [32, 46] and in three studies using the Functional Assessment of Cancer Therapy‐Prostate (FACT‐P) tool [30, 42, 50].

Fatigue was assessed in five studies using the Functional Assessment of Cancer Therapy‐Fatigue (FACT‐F) [30, 32, 42, 46, 47], and only one study used the Multidimensional Fatigue Symptom Inventory‐Short Form (MSFI‐SF) [31].

One study evaluated bone pain using the Visual Analogue Scale (VAS) and the Functional Assessment of Cancer Therapy‐Bone Pain (FACT‐BP) [31].

Psychological distress was assessed in three studies using the Brief Symptom Inventory 18 (BSI‐18) tool [31, 32, 45].

Finally, intervention‐related adverse events were reported in three studies [39, 47, 48].

### 3.3 Risk of Bias in Included Studies

The overall risk of bias was considered high in 8 of the 18 RCTs [33, 36, 38, 39, 40, 41, 43, 46]. Four RCTs presented some concerns regarding the risk of bias [30, 31, 37, 42], and six RCTs were considered as having a low overall risk of bias [32, 33, 45, 47, 48, 49]. One of the LOS presented a moderate risk of bias [44] whereas the other was rated at critical risk of bias [35]. The detailed judgements for each of the risk of bias domain criteria are shown in Table 2.

**TABLE 2:** Risk of bias assessment.

RoB 2.0						
Study	Domains					
	Random process	Deviations from intended interventions	Missing outcome data	Measurement of the outcome	Selection of the reported result	Overall
Bourke, 2014 [30]	Low	Low	Some concerns	Low	Low	Some concerns
Cormie, 2013 [31]	Low	Low	Low	Low	Some concerns	Some concerns
Cormie, 2015 [32]	Low	Low	Low	Low	Low	Low
Focht, 2018 [48]	Low	Low	Low	Low	Low	Low
Galvao, 2010 [33]	Low	Low	Low	Low	Low	Low
Galvao, 2018 [34]	Low	High	Some concerns	Low	Low	High
Hojan, 2017 [46]	Low	Some concerns	Low	Low	Some concerns	High
Houben 2023 [36]	Some concerns	High	Low	Low	Low	High
Lam, 2019 [37]	Some concerns	Low	Low	Low	Low	Some concerns
Ndjavera, 2020 [47]	Low	Low	Low	Low	Low	Low
Nilsen, 2015 [49]	Low	Low	Low	Low	Low	Low
Nilsen, 2016 [38]	Some concerns	Some concerns	Low	Low	Low	High
Padhi, 2014 [39]	Some concerns	High	High	Low	Some concerns	High
Rhee, 2021 [40]	Some concerns	High	High	Low	Low	High
Sajid, 2016 [41]	High	High	High	Low	Some concerns	High
Segal, 2003 [42]	Low	Low	Low	Low	Some concerns	Some concerns
Uth, 2014 [43]	Low	High	Some concerns	Low	Low	High
Wall, 2017 [45]	Low	Low	Low	Low	Low	Low

ROBINS‐I							
Study	Domains						
	Bias due to confounding	Bias in selection of participants	Bias in classification of interventions	Bias due to deviations from intended interventions	Bias due to missing data	Bias in measurement of outcomes	Bias in selection of the reported result
Hong, 2013 [35, p. 20]	Low	Critical	Serious	NI	Low	Low	Critical
Uth, 2018 [44]	NI	Low	Moderate	Low	Low	Low	Moderate

*Note:* High: high risk of bias; low: low risk of bias; some concerns: some concerns about the risk of bias.

### 3.4 Synthesis of Results

#### 3.4.1 Survival

One RCT evaluating the use of MA reported no statistically significant differences in CSS between the low‐dose MA group, high‐dose MA group and control group (very low CoE) (Table S3) [35].

#### 3.4.2 Muscle Mass

Only one RCT [30], assessing an LSI combining exercise and diet, reported on skeletal muscle mass, which was measured by bioelectrical impedance. A statistically significant difference in skeletal muscle mass was found between the intervention and control groups (adjusted MD [95% CI] = 0.6 kg [0.1, 1.1]; *p* = 0.03; Moderate CoE).

**FIGURE 2:**
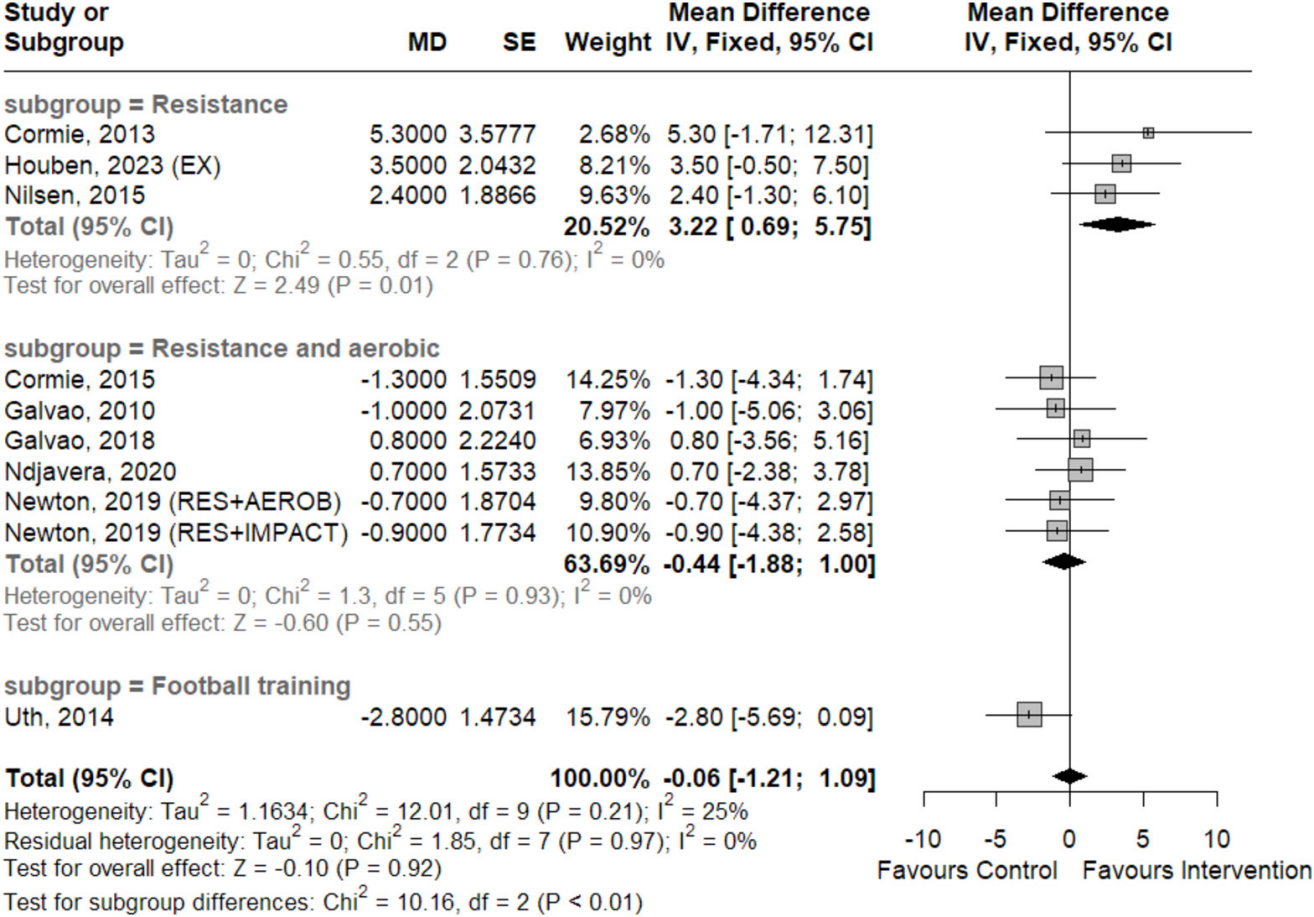
Forest plot for the effects of ET on lean body mass (kg).

Ten RCTs incorporated exercise training (ET) as an intervention for lean mass‐loss prevention [31, 32, 33, 34, 36, 41, 43, 45, 47, 49]. No clear effect on lean body mass was observed (random effects, MD [95% CI] = −1.29 kg [−4.06, 1.48]; *p* = 0.36; *k* = 10), and heterogeneity was markedly high (*I* ^2^ = 71%; *p* < 0.01). However, after excluding the study by Sajid et al. [41] during the sensitivity analysis, which showed an anomalous lean body mass increase for the control group, and conducting a subgroup analysis by the type of ET performed, heterogeneity disappeared and the resistance exercise subgroup demonstrated a statistically significant effect, which can be seen in Figure 2 below (CoE was moderate for this outcome measure).

An RCT assessing a resistance ET programme [37] could not be included in the meta‐analysis as it did not report all the required information. However, this study reported a statistically significant difference in the prevention of lean body mass loss in the resistance training group compared with control (MD [95% CI] = 1.1 kg [0.39, 1.81]; *p* < 0.01).

One LOS also implemented football training for 5 years [44], but no statistically significant results were found for lean body mass (MD [95% CI] = −1.1 kg [−5.13, 2.93]; *p* = 0.59).

ET + PRO showed an increase in skeletal muscle mass (quadriceps cross‐sectional area) compared with usual care in a single RCT (MD ± SD = 1.9 ± 2.7 cm2; *p* < 0.001; low CoE) [36]. One RCT assessing an LSI consisting of GMCB‐RET and dietary advice reported no statistically significant difference compared with the control group (MD [95% CI] = 0.44 [−0.6, 1.48]; *p* = 0.41; moderate CoE) [48].

Furthermore, two RCTs assessing pharmacological interventions reported on lean body mass. Liver targeted oral testosterone therapy [40] showed effectiveness in preventing the loss of lean body mass compared with placebo (MD [95% CI] = 0.8 [−0.1, 1.7]; *p* = 0.04; low CoE) and AMG745 [39] showed effectiveness in preventing both the loss of lean body mass (MD [SE] = 2.2% [0.8%]; *p* = 0.008; low CoE) and lower‐extremity muscle size (MD [SE] = 1.8% [1%]; *p* = 0.065; low CoE) compared with placebo.

#### 3.4.3 Muscle Strength

Seven RCTs assessing ET programmes reported on lower‐body strength and were included in the meta‐analysis [31, 32, 33, 34, 43, 45, 49].

**FIGURE 3:**
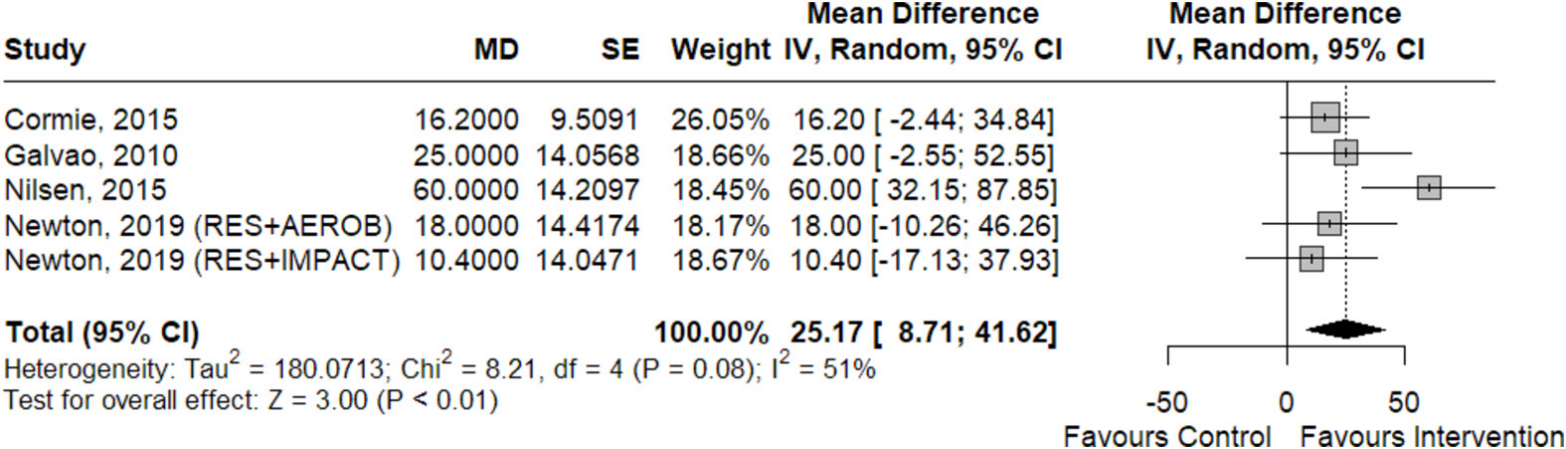
Forest plot for the effects of ET on 1RM leg press (kg).

**FIGURE 4:**
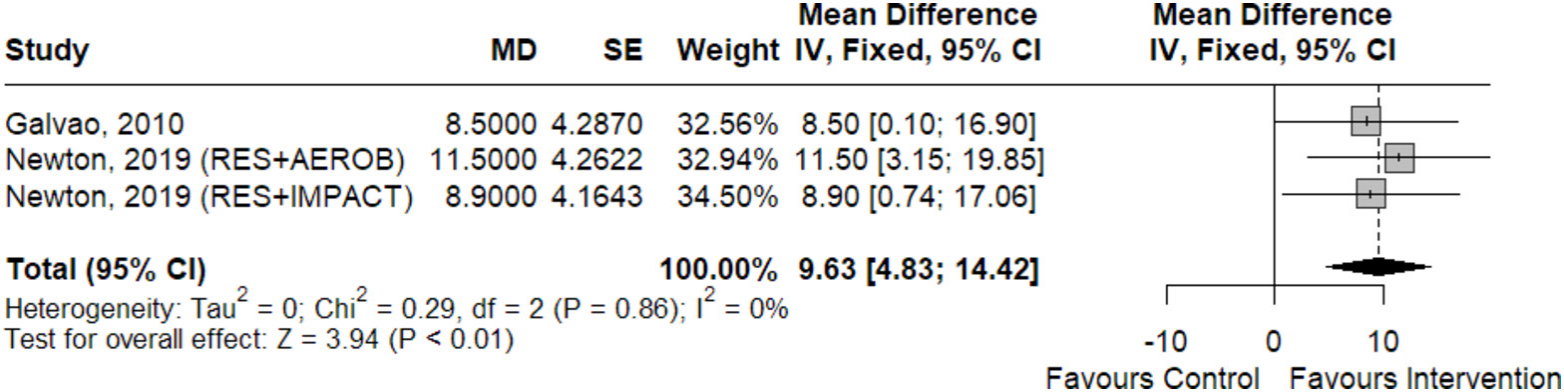
Forest plot for the effects of ET on 1RM leg extension (kg).

Among these studies, four reported on leg press [32, 33, 45, 49], three on knee extension [31, 34, 43] and two on leg extension strength [33, 45], and they were meta‐analysed. A statistically significant difference in muscle strength was observed for 1RM leg press weight lifted (low CoE) (Figure 3) and leg extension weight lifted (moderate CoE) (Figure 4). However, no significant differences were found for knee extension (fixed effects, MD [95% CI] = 3.41 kg [−3.53, 34.40]; *p* = 0.96; *k* = 3; low CoE).

Two RCTs, which also implemented ET programmes, could not be included in the meta‐analysis as they reported performance modifications through weight percentage change [36, 38]. In Nilsen et al., a significantly larger increase in 1RM knee extension weight lifted was found in the ET group compared with the control group (MD [95% CI] = 19.00% [11.18, 26.82]; *p* < 0.001) [38]. Houben et al. reported significant increases for the ET group in the change in 1RM leg press (MD [95% CI] = 17.00% [9.64, 24.36]; *p* < 0.001) and 1RM leg extension weight lifted (MD [95% CI] = 25.00% [15.90, 34.10]; *p* < 0.001) compared with the control group. The ET + PRO group also showed statistically significant increases in the change in 1RM leg press (MD [95% CI] = 18.00% [11.10, 24.90]; *p* ≤ 0.001; low CoE) and 1RM leg extension weight lifted performance (MD [95% CI] = 21.00% [10.65, 31.35]; *p* ≤ 0.001; low CoE) compared with control [36]. Additionally, one LOS also reported on 1RM knee extension (kg), but no clear significant effect was observed (MD [95% CI] = −2.4 kg [−17.0, 12.2]; *p* = 0.732) [44].

One RCT assessing an LSI consisting of GMCB‐RET and dietary advice reported a statistically significant difference in 1RM leg extension strength change, compared with control (MD [95% CI] = 11.14 [1.64, 20.64]; *p* = 0.02; moderate CoE) [48].

**FIGURE 5:**
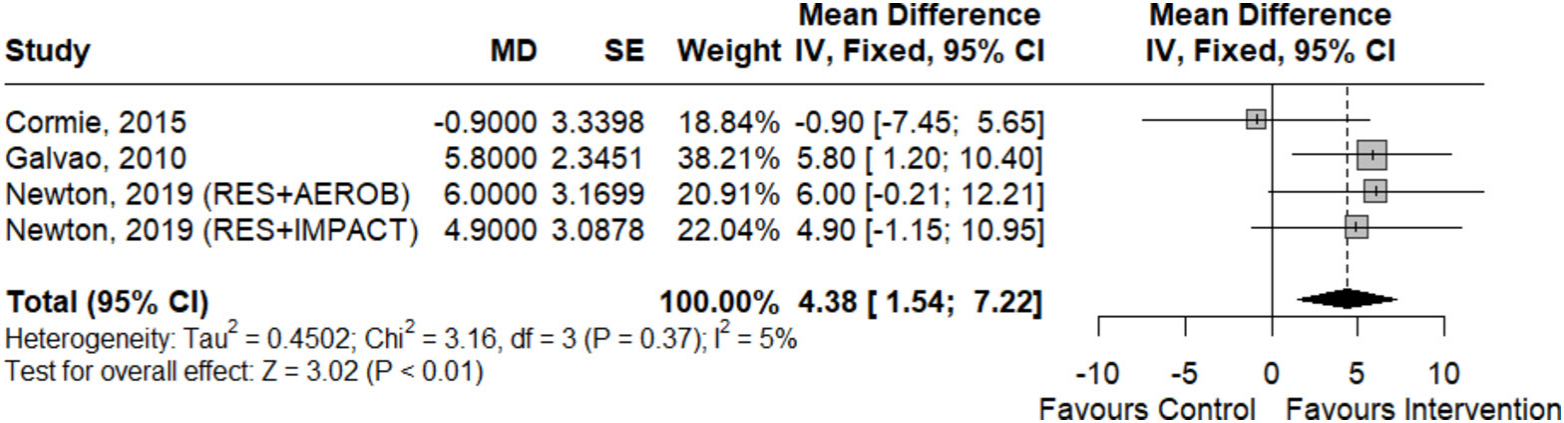
Forest plot for the association between exercise training and 1RM seated row (kg).

On the other hand, six RCTs evaluating ET interventions reported on upper‐body strength and were included in the meta‐analysis [32, 33, 41, 45, 49]. A significant effect was found for seated row (moderate CoE) (Figure 5) [32, 33, 45]. However, no significant results were found for chest press (fixed effects, MD [95% CI] = 1.70 kg [−1.48, 4.88]; *p* = 0.30; *k* = 4; moderate CoE) [32, 33, 45], shoulder press (MD [95% CI] = −5 kg [−0.47, 10.47]; *p* = 0.07; moderate CoE) [49] or handgrip strength (random effects, MD [95% CI] = 0.34 kg [−5.66, 6.34]; *p* = 0.91; *k* = 3; *I* ^2^ = 68%; very low CoE) [41]. The study by Sajid et al. was excluded from the chest press analysis due to anomalous data reported [41]. GMCB‐RET and dietary advice LSI showed a significant effect compared with control in the change in 1RM chest press strength change (MD [95% CI] = 9.36 [0.83, 17.89]; *p* = 0.03; moderate CoE).

#### 3.4.4 Physical Functioning

Four RCTs evaluating the effect of ET programmes assessed physical functioning using various walking tests and were included in the meta‐analysis [32, 33, 34, 57]. No statistically significant effects were found in 400‐m walk (fixed effects, MD [95% CI] = −7.48 s [−17.49, 2.54]; *p* = 0.14; *k* = 5; moderate CoE) [32, 33, 34, 57], 6‐m walk at usual pace (random effects, MD [95% CI] = −0.03 s [−0.35, 0.29]; *p* = 0.85; *k* = 3; *I* ^2^ = 60%; low CoE) or fast pace (fixed effects, MD [95% CI] = −0.04 [−0.19, 0.12]; *p* = 0.64; *k* = 3; moderate CoE) and in steps walked per day (random effects, MD [95% CI] = 3348.64 [−1510.27, 8207.55]; *p* = 0.14; *k* = 2; *I* ^2^ = 55%; very low CoE).

The study by Bourke et al., which evaluated an LSI combining ET and dietary advice, used a treadmill walk test to assess exercise tolerance. The LSI group showed significant improvement in exercise tolerance compared with control (MD [95% CI] = 121.2 s [91.2, 151.2]; *p* < 0.001; moderate CoE) [30]. The study by Focht et al., which assessed a GMCB‐RET and dietary advice LSI, reported a significant effect in 400‐m walk (MD [95% CI] = −78.67 s [−156.12, −1.22]; *p* = 0.05; moderate CoE) [48].

Physical functioning was also assessed by various individual functional tests in seven RCTs evaluating the effect of ET programmes [32, 33, 34, 36, 43, 45, 49]. Statistically significant effects were found for chair sit‐to‐stand test measured through the number of seconds taken to perform five repetitions (fixed effects, MD [95% CI] = −1.02 s [−1.70, −0.34]; *p* < 0.01; *k* = 4; moderate CoE) [32, 33, 45] and by the number of repetitions in 30 s (fixed effects, MD [95% CI] = 1.43 [0.22, 2.65]; *p* = 0.02; *k* = 3; low CoE) [36, 43, 49]. No significant effects were reported for timed up‐and‐go test (fixed effects, MD [95% CI] = 0.50 s [−0.17, 1.16]; *p* = 0.14; *k* = 2; low CoE) [34, 38]; stair climb (fixed effects, SMD [95% CI] = −0.12 [−0.37, 0.12]; *p* = 0.33; *k* = 5; moderate CoE) [32, 33, 36, 43, 49] and jump height (MD [95% CI] = 1.00 [−1.53, 3.53]; *p* = 0.44; low CoE) [43]. The study by Sajid et al. found no significant effect on the SPPB score when comparing two different ET programmes to control (fixed effects, MD [95% CI] = 3.04 [−0.38, 6.45]; *p* = 0.08; *k* = 2; low CoE) [41].

Houben et al. reported no significant results in the chair sit‐to‐stand, timed up‐and‐go and stair climb tests for the ET + PRO group compared with the control group (low CoE) (Table S3) [36].

Bourke et al. also performed a chair sit‐to‐stand test and found a significant increase of repetitions in the LSI group compared with control (adjusted MD [95% CI] = 3.79 [1.68, 5.90]; *p* = 0.002; moderate CoE) [30]. Focht et al. found no significant improvement in stair climb test time in the LSI group compared with control (MD [95% CI] = −4.33 [−9.64, 0.98]; *p* = 0.11; moderate CoE) [48].

#### 3.4.5 Balance

Four RCTs assessing ET programmes tested balance with different tests and were included in the meta‐analysis [32, 33, 34, 43]. No significant changes were observed in 6‐m backwards walk (fixed effects, MD [95% CI] = −1.25 s [−3.89, 1.38]; *p* = 0.35; *k* = 4; moderate CoE) [32, 33, 45]; SOT score (fixed effects, MD [95% CI] = −0.84 [−4.07, 2.38]; *p* = 0.61; *k* = 3; moderate CoE) [32, 33, 34] or ABC score (fixed effects, MD [95% CI] = −0.89 [−11.04, 9.26]; *p* = 0.86; *k* = 2; moderate CoE) [32, 33].

Uth et al. also assessed balance by the flamingo balance test and bipedal and tandem stance sway areas, although no significant results were found for the ET group compared with the control group [43] (low CoE) (Table S3).

#### 3.4.6 HRQOL

Three RCTs assessing ET reported on general HRQoL using the SF‐36 tool and were meta‐analysed. No significant effects were found for the physical (fixed effects, MD [95% CI] = −1.28 [−4.62, 2.05]; *p* = 0.45; *k* = 2; moderate CoE) [31, 32] and mental (fixed effects, MD [95% CI] = 3.52 [−0.25, 7.28]; *p* = 0.07; *k* = 2; moderate CoE) [31, 32] health composites or for the physical functioning domain (random effects, MD [95% CI] = 1.96 [−3.33, 7.25]; *p* = 0.47; *k* = 2; *I* ^2^ = 78%; very low CoE) [32, 34].

Two RCTs assessing ET reported HRQoL using the QLQ‐C30 tool [46, 49]. Significant improvements were found in the ET group for the cognitive (fixed effects, MD [95% CI] = 7.78 [1.08, 14.47]; *p* = 0.02; *k* = 2; low CoE), emotional (fixed effects, MD [95% CI] = 9.25 [3.02, 15.49]; *p* < 0.01; *k* = 2; low CoE) and physical domains (fixed effects, MD [95% CI] = 5.65 [0.02, 11.28]; *p* = 0.05; *k* = 2; low CoE) when compared with control.

One ET RCT also assessed HRQoL using the FACT‐G tool and reported a significant improvement in the ET group when compared with the control group (MD [95% CI] = 17.90 [15.11, 20.69]; *p* = 0.01; low CoE) [46].

An RCT assessing an LSI also used the FACT‐G tool to assess HRQoL and found no significant effect in general HRQoL [30] (moderate CoE) (Table S3).

Three RCTs assessing ET programmes reported on PCa‐specific HRQoL using the FACT‐P [42] and QLQ‐PR25 [32, 46] tools. A significant effect was only found for the sexual function domain of the QLQ‐PR25, showing an increased score for the ET group compared with the control group (MD [95% CI] = 16.92 [10.41, 23.44]; *p* < 0.01; *k* = 2; low CoE), as well as for the treatment‐related symptoms, which decreased significantly in the ET group compared with the control group (MD [95% CI] = −4.52 [−8.99, −0.05]; *p* = 0.05; *k* = 2; low CoE) [32, 46].

Regarding LSI compared with control, one RCT found no significant effect in PCa‐specific HRQoL using the FACT‐P (Moderate CoE) (Table S3) [30].

#### 3.4.7 Fatigue

Four RCTs on ET assessed fatigue using the FACT‐F tool. Heterogeneity was very high and meta‐analysis showed no significant effect (random effects, MD [95% CI] = 6.70 [−1.86, 15.25]; *p* = 0.13; *k* = 4; *I* ^2^ = 97%) [31, 32, 45, 46]. After removing the study by Hojan et al., heterogeneity disappeared, but no statistically significant effect was found either (fixed effects, MD [95% CI] = 1.92 [−0.40, 4.23]; *p* = 0.11; *k* = 3; *I* ^2^ = 0%; moderate CoE) [31, 32, 45]. Another RCT on ET assessed fatigue using the MSFI‐SF tool and reported no significant effects either (MD [95% CI] = 5.00 [−12.61, 22.61]; *p* = 0.58; low CoE).

#### 3.4.8 Psychological Distress

Three RCTs on ET evaluated psychological distress through the BSI‐18 tool and were meta‐analysed [31, 32, 45]. Statistically significant improvements were obtained for the ET group in the somatization domain (fixed effects, MD [95% CI] = −0.69 [−1.32, −0.07]; *p* = 0.03; *k* = 4; moderate CoE) and in the global severity index (fixed effects, MD [95% CI] = −1.63 [−3.10, −0.15]; *p* = 0.03; *k* = 4; moderate CoE) domains of the BSI‐18 compared with the control group.

#### 3.4.9 Adverse Events

Three RCTs reported on intervention‐related adverse events [39, 47, 48]. The study by Ndjavera et al., which assessed an ET intervention, reported no adverse events during training (moderate CoE) [47]. Focht et al., who assessed a GMCB‐RET and dietary advice LSI, reported only one case of musculoskeletal pain in the shoulder and back, which required a modification of upper‐body exercises and resolved without additional treatment (moderate CoE). The study by Padhi et al., whose intervention focused on different doses of AMG745, reported only mild or moderate non‐serious adverse events (fatigue, injection site bruising, diarrhoea and contusion), except for one serious adverse event of syncope (low CoE) [39].

#### 3.4.10 Publication Bias Assessment

**FIGURE 6:**
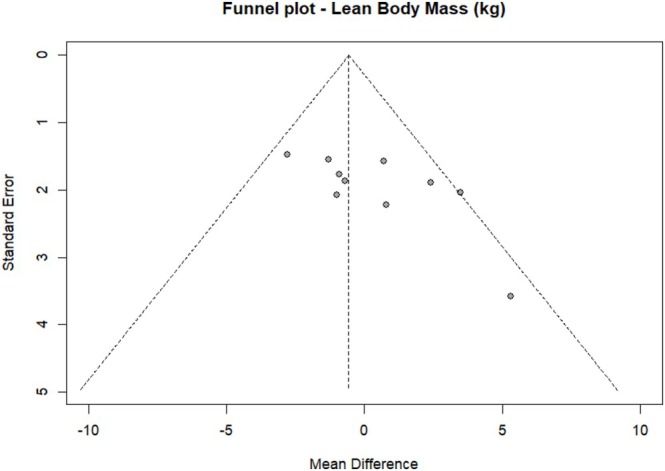
Funnel plot for the effect of ET on lean body mass (kg).

The number of studies reporting most of the outcome measures evaluated was too small (*k* = 1 to 5) to perform any valid publication bias assessment, as there was insufficient information to assess asymmetry or trends that could indicate bias reliably. Therefore, publication bias was only assessed for lean body mass, measured in studies that assessed ET programmes (*k* = 10). No apparent asymmetries were observed, but Figure 6 shows a certain absence of small studies reporting results favouring the control groups, which could indicate a possible small‐study effect in the meta‐analysis. The Egger test showed a coefficient of −2.35 (95% CI [−5.59, −0.88]; *p* = 0.18), indicating no statistically significant asymmetry. The study by Sajid et al. was excluded from the publication bias assessment because, as mentioned above, it showed anomalous data for this outcome measure [41]. All funnel plots are available in Data S2.

## 4 Discussion

This review identified 20 studies [30, 31, 32, 33, 34, 35, 36, 37, 38, 39, 40, 41, 42, 43, 44, 45, 46, 47, 48, 49], including RCTs and LOSs, assessing interventions such as physical exercise programmes, lifestyle modifications and pharmacological treatments.

Resistance ET demonstrated a statistically significant preventive effect on lean body mass loss in men undergoing ADT [31, 32, 33, 34, 36, 43, 45, 47, 49]. The use of AMG745 [39] and oral testosterone [40] also showed a significant preventive effect of lean body mass loss. Regarding muscle strength, significant improvements were observed with ET in lower‐body strength measures such as 1RM leg press [32, 33, 45, 49] and leg extension [33, 45]. An LSI also showed significant improvements in 1RM leg extension [48]. Upper‐body strength gains were less consistent, being only significant for seated row strength during ET [32, 33, 45] and for chest press on an LSI [48].

Regarding physical functioning, ET showed significant improvements in chair sit‐to‐stand tests measured in time [32, 33, 45] and repetitions [36, 43, 49]. An LSI showed significant improvements in exercise tolerance [30] and another LSI in 400‐m walk time [48].

ET also improved some general and disease‐specific HRQoL domains, such as the cognitive, emotional and physical domains of the QLQ‐C30 tool [46, 49], the overall FACT‐G score [46] and the sexual function domain of the QLQ‐25 [32, 46]. ET also showed significant effects in the somatization and global severity index domains of the BSI‐18 psychological distress tool [31, 32, 45]. Finally, reported intervention‐related adverse events of interventions were generally mild and non‐serious [39, 47, 48].

Nutritional interventions were implemented only as complementary components of multicomponent lifestyle interventions, rather than as standalone strategies and consisted of dietary advice aimed at improving overall diet quality [30, 48]. Only one study assessing protein supplementation clearly defined the nutritional intervention performed [36]. Consequently, the available evidence does not allow firm conclusions regarding the independent effects of nutritional interventions on muscle‐related or functional outcomes in this population. Future studies should evaluate well‐defined nutritional strategies, including macronutrient‐based and supplementation approaches, both as standalone interventions and in combination with exercise, to better clarify their role in the prevention and management of muscle loss in patients with advanced PCa.

The findings here are consistent with those of previous systematic reviews [59, 60, 61], which highlight the benefits of resistance exercise on muscle mass, strength and quality of life. However, the present work offers a broader scope, including not only exercise interventions but also lifestyle modifications and pharmacological treatments. This comprehensive approach provides a holistic understanding of the potential strategies for managing sarcopenia in patients with advanced prostate carcinoma undergoing ADT. Nevertheless, the present scarcity of evidence on supplementation and dietary interventions for the prevention or management of sarcopenia in patients with advanced PCa—which may offer additional benefits when combined with physical exercise—is particularly relevant. This underscores the urgent need for further research in this domain, as it represents a potential cornerstone in the prevention and management of this condition.

The main limitation of the present review is that the included studies did not report on the main outcome measures of survival and sarcopenia. Instead, they primarily focused on measuring muscle or lean mass, muscle strength and physical functioning. The absence of these outcome measures hinders a complete understanding of the long‐term impact of interventions on sarcopenia and survival outcomes in this population.

Most of the included studies were characterized by small sample sizes. In addition, due to the lack of available data, certain subgroup analyses could not be performed to explore the impact of important patient variables like age, presence of metastases or sarcopenia stage and intervention characteristics such as type and duration of exercise interventions on the magnitude of association.

The synthesis of results was complicated by the heterogeneity across the studies. Variations in the type of interventions, outcome measures and assessment tools reduced the statistical power of the meta‐analyses, preventing definitive conclusions about the effectiveness of specific interventions to be drawn.

To address these limitations, future research should focus on conducting larger RCTs with extended intervention periods and follow‐up durations to capture long‐term effects on sarcopenia and mortality. Furthermore, systematic physical functionality assessment methods, such as standardized test batteries such as the Short Physical Performance Battery (SPPB) should be implemented to ensure consistency and comparability across studies. These standardized measures would facilitate more robust meta‐analyses and clearer conclusions.

Furthermore, this review acknowledges the possibility that some relevant studies might not have been included, either because they were not available in English or Spanish or were not indexed in the searched databases.

Despite its limitations, the present study benefits from rigorous methods, adhering to the fundamental principles of transparency and replicability in systematic reviews. These include a comprehensive search, duplicate processes of study selection, data extraction and risk of bias assessment, along with a quantitative synthesis of results and an assessment of the CoE approached in a structured and explicit manner.

The present systematic review highlights the potential benefits of various interventions in managing sarcopenia in patients with advanced PCa undergoing ADT. However, the significant heterogeneity and lack of comprehensive outcome reporting underscore the need for more standardized and long‐term research to draw conclusive evidence. Future studies should prioritize larger, well‐designed RCTs with standardized measurement methods to enhance the reliability and applicability of findings in clinical practice.

## Funding

The authors report no specific funding for the study.

## Consent

The authors have nothing to report.

## Conflicts of Interest

The authors declare no conflicts of interest.

## Supporting information


**Data S1:** Supplementary Information.

**Data S2:** Supplementary Information.

**Table S1:** Search strategy.

**Table S2:** Excluded studies

**Table S3:** Results of all meta‐analysis and subgroup analysis of exercise training (ET) interventions and analysis not reported in the manuscript regarding other interventions

## Data Availability

The data presented in this study are available in the Supporting Information.
