# Supplementary material for: Effectiveness and Safety of Interventions for Sarcopenia in Advanced Prostate Carcinoma: Systematic Review
Source: J Cachexia Sarcopenia Muscle. 2026 May 5;17(3):e70290. doi: 10.1002/jcsm.70290 (PMC13144553; doi:10.1002/jcsm.70290)
Supplement: Supplementary file 3 — Table S1: Search strategy. [file JCSM-17-e70290-s001.docx]

Supplementary Table S1. Search strategy.

| **MEDLINE** |
| --- |
| 1. exp Prostatic Neoplasms/ 2. (Prostat* adj5 (Neo?plasm$ or cancer or tumo?r or carcinom* or adenoma* or adenocarcin* or mass or masses or cyst* or oncolog* or sarcom* or malignan$*)).tw. 3. prostat*.hw. and exp Neoplasms/ 4. 1 or 2 or 3 5. "Aged, 80 and over"/ or Aged/ 6. Frail Elderly/ 7. ((("60" or "65" or 60-65) adj (elder* or adult* or aged or age or old or older or over or year*)) or elder* or adult*).mp. 8. 5 or 6 or 7 9. 4 and 8 10. Sarcopenia/ 11. (muscle* adj4 (deplet* or loss or wasting or reduction or skeletal or attenuation or strength or fatigue or atrophy)).ti,ab. 12. sarcopen*.ti,ab,kw. 13. exp Cachexia/ 14. cache*.ti,ab. 15. 10 or 11 or 12 or 13 or 14 16. 9 and 15 17. limit 16 to (english language or spanish) 18. limit 17 to humans |
| **Embase** |
| 1. 'prostate tumor'/exp 2. (prostat* NEAR/5 (neo?plasm* OR cancer OR tumo?r OR carcinom* OR adenoma* OR adenocarcin* OR mass OR masses OR cyst* OR oncolog* OR sarcom* OR malignan$*)):ti,ab,de 3. prostat*:ti,ab,de AND 'neoplasm'/exp 4. #1 OR #2 OR #3 5. 'aged'/exp 6. 'very elderly'/exp 7. 'frail elderly'/exp 8. ((('60' OR '65' OR '60-65') NEAR/1 (elder* OR adult* OR aged OR age OR old OR older OR over OR year*)):ti,ab,de) OR elder*:ti,ab,de OR adult*:ti,ab,de 9. #5 OR #6 OR #7 OR #8 10. 'sarcopenia'/exp 11. (muscle* NEAR/4 (deplet* OR loss OR wasting OR reduction OR skeletal OR attenuation OR strength OR fatigue OR atrophy)):ti,ab 12. sarcopen*:ti,ab,de 13. 'cachexia'/exp 14. cache*:ti,ab 15. #10 OR #11 OR #12 OR #13 OR #14 16. #4 AND #9 AND #15 17. #4 AND #9 AND #15 AND ([english]/lim OR [spanish]/lim) 18. #4 AND #9 AND #15 AND ([english]/lim OR [spanish]/lim) AND [humans]/lim |
| **WOS** |
| 1. TS= (Prostat* NEAR/5 (Neo?plasm* or cancer or tumo?r or carcinom* or adenoma* or adenocarcin* or mass or masses or cyst* or oncolog* or sarcom* or malignan*)) 2. TS= ((("60" or "65" or 60-65) NEAR/1 (elder* or adult* or aged or age or old or older or over or year*)) or elder* or adult*) 3. TS= sarcopen* 4. TS= (muscle* NEAR/4 (deplet* or loss or wasting or reduction or skeletal or attenuation or strength or fatigue or atrophy)) 5. TS= cache* 6. #5 OR #4 OR #3 7. #6 AND #2 AND #1 |
