# Supplementary material for: Effectiveness and Safety of Interventions for Sarcopenia in Advanced Prostate Carcinoma: Systematic Review
Source: J Cachexia Sarcopenia Muscle. 2026 May 5;17(3):e70290. doi: 10.1002/jcsm.70290 (PMC13144553; doi:10.1002/jcsm.70290)
Supplement: Supplementary file 4 — Table S2: Excluded studies [file JCSM-17-e70290-s003.docx]

| Supplementary Table S2. Excluded studies |
| --- |
| Abstract |
| 1. Bhargava et al., Role of nutritional supplement ensure protein max on calorie and protein intake, appetite and body weight in patients with advanced cancer receiving chemotherapy. Supportive Care in Cancer. 2019; 27 (1): 5141. 2. Bubley, G et al., Phase II study of enzalutamide monotherapy with radiation therapy for intermediate risk prostate cancer. JCO. 2018; 36: 58-58. doi:10.1200/JCO.2018.36.6_suppl.58. 3. Bylow KA et al., Effect of β-hydroxy-β-methylbutyrate (HMB) on muscle strength in older men with prostate cancer (Pca) started on androgen deprivation therapy (ADT): Preliminary results of an open-label, randomized trial. JCO. 2018; 36: 258-258. doi:10.1200/JCO.2018.36.6_suppl.258. 4. Dorff TB et al., Impact of resistance exercise on metabolic syndrome (MetS) parameters in men receiving androgen deprivation therapy (ADT) for prostate cancer. JCO. 2017; 35: 223-223. doi:10.1200/JCO.2017.35.6_suppl.223 5. Dorff TB et al., Impact of resistance exercise on metabolic syndrome (MetS) parameters in men receiving androgen deprivation therapy (ADT) for prostate cancer. Annals of Oncology. 2017; 28 (supp. 5): V555. 6. Farley M et al., The interplay between inflammatory markers and body composition with 6 months of HIIT in breast, colorectal and prostate cancer survivors. Asia-Pacific Journal of Clinical Oncology. 2020; 16 (suppl. 8): 203. 7. Farley M et al., Changes in body composition and sarcopenia status with four weeks of HIIT in breast and prostate and colorectal cancer survivors. Asia-Pacific Journal of Clinical Oncology. 2020; 16 (suppl. 8): 119. 8. Fraser S. Exercise and nutrition to treat adverse musculoskeletal effects of hormone therapy in prostate cancer. Asia Pac J Clin Oncol. 2019; 15 (suppl. 9): 67-68. 9. Inglis J et al., 10. Karzai F et al., A phase 2 study of olaparib and durvalumab in metastatic castrate-resistant prostate cancer (mCRPC) in an unselected population. J Clin Oncol. 2018; 36 (6). 11. Kilari D et al., Role of comprehensive geriatric assessment (CGA) in identifying elderly patients with systemic prostate cancer (PCa) for peripheral androgen blockade (PAB). Journal of the American Geriatrics Society. 2013; 61: 532. 12. Kiwata JL et al., Effect of a supervised exercise intervention on sarcopenic obesity and metabolic syndrome in prostate cancer patients: A randomized pilot study. Cancer Res. 2017; 77 (13). 13. Mitsui Y et al., Impact of sarcopenia on longitudinal erectile functional outcomes after nerve-sparing robot assisted radical prostatectomy. Eu Uro Op Sci. 2020; 19: e1112-e1113. 14. Naimi MF et al., Comparative analysis of sarcopenia induced by long-term abiraterone (A) versus enzalutamide (E) therapy in men with metastatic castrationresistant prostate cancer (mCRPC). J Clin Oncol. 2021. 39 (suppl. 6). 15. Newton RU et al. Exercise medicine to arrest bone loss in men with prostate cancer undergoing androgen deprivation therapy: A 12-month randomized controlled trial. BJU Int. 2017; 120. 16. Olesen RD et al., Effects of 12 weeks of supervised endurance and strength training on muscle strength, physical fitness and body composition in prostate cancer patients undergoing Androgen Deprivation Therapy. Scand J Urol. 2019; 53: 34. 17. Rhee H et al., A novel liver-targeted testosterone therapy for sarcopaenia in androgen deprived menwith prostate cancer: a double-blind placebo-controlled study. Asia Pac J Clin Oncol. 2019. 15: 30. 18. Singh F et al., Supervisedon-site exercise during acute radiation treatment: Effect on muscle strength, physical function and body composition. Asia Pac J Clin Oncol. 2017. 13: 74-75. 19. Taaffe DR et al., Immediate versus delayed exercise in men initiating androgen deprivation: Effects on bone density and tissue composition. BJU Int. 2017; 120: 15 20. Thekkekara RJ et al., Lean and fat-mass changes following upfront docetaxel compared to androgen deprivation monotherapy in metastatic castration-na+»ve prostate cancer. J Clin Oncol. 2018; 36: 15. 21. Wilson RL et al., Contrasting exercise modes enhance muscle strength and physical function in prostate cancer patients undertaking androgen deprivation therapy: A 12-month randomized controlled trial. BJU Int. 2017; 120: 15-16. |
| **Population** |
| 1. Alberga AS, Segal RJ, Reid RD, Scott CG, Sigal RJ, Khandwala F, Jaffey J, Wells GA, Kenny GP. Age and androgen-deprivation therapy on exercise outcomes in men with prostate cancer. Support Care Cancer. 2012 May; 20(5):971-81. doi: 10.1007/s00520-011-1169-x. 2. Cheung AS, de Rooy C, Levinger I, Rana K, Clarke MV, How JM, Garnham A, McLean C, Zajac JD, Davey RA, Grossmann M. Actin alpha cardiac muscle 1 gene expression is upregulated in the skeletal muscle of men undergoing androgen deprivation therapy for prostate cancer. J Steroid Biochem Mol Biol. 2017 Nov; 174:56-64. doi: 10.1016/j.jsbmb.2017.07.029. 3. Frawley HC, Lin KY, Granger CL, Higgins R, Butler M, Denehy L. An allied health rehabilitation program for patients following surgery for abdomino-pelvic cancer: a feasibility and pilot clinical study. Support Care Cancer. 2020 Mar; 28(3):1335-1350. doi: 10.1007/s00520-019-04931-w. 4. LaStayo PC, Marcus RL, Dibble LE, Smith SB, Beck SL. Eccentric exercise versus usual-care with older cancer survivors: the impact on muscle and mobility--an exploratory pilot study. BMC Geriatr. 2011 Jan 27; 11:5. doi: 10.1186/1471-2318-11-5. 5. Monga U, Garber SL, Thornby J, Vallbona C, Kerrigan AJ, Monga TN, Zimmermann KP. Exercise prevents fatigue and improves quality of life in prostate cancer patients undergoing radiotherapy. Arch Phys Med Rehabil. 2007 Nov; 88(11):1416-22. doi: 10.1016/j.apmr.2007.08.110. 6. Mustian KM, Peppone L, Darling TV, Palesh O, Heckler CE, Morrow GR. A 4-week home-based aerobic and resistance exercise program during radiation therapy: a pilot randomized clinical trial. J Support Oncol. 2009 Sep-Oct; 7(5):158-67. 7. Ottenbacher A, Yu M, Moser RP, Phillips SM, Alfano C, Perna FM. Population Estimates of Meeting Strength Training and Aerobic Guidelines, by Gender and Cancer Survivorship Status: Findings From the Health Information National Trends Survey (HINTS). J Phys Act Health. 2015 May; 12(5):675-9. doi: 10.1123/jpah.2014-0003. 8. Papadopoulos E, Gillen J, Moore D, Au D, Kurgan N, Klentrou P, Finelli A, Alibhai SMH, Santa Mina D. High-intensity interval training or resistance training versus usual care in men with prostate cancer on active surveillance: a 3-arm feasibility randomized controlled trial. Appl Physiol Nutr Metab. 2021 Dec; 46(12):1535-1544. doi: 10.1139/apnm-2021-0365. 9. Singh F, Newton RU, Baker MK, Spry NA, Taaffe DR, Thavaseelan J, Galvão DA. Feasibility of Presurgical Exercise in Men With Prostate Cancer Undergoing Prostatectomy. Integr Cancer Ther. 2017 Sep; 16(3):290-299. doi: 10.1177/1534735416666373. 10. Storck LJ, Ruehlin M, Gaeumann S, Gisi D, Schmocker M, Meffert PJ, Imoberdorf R, Pless M, Ballmer PE. Effect of a leucine-rich supplement in combination with nutrition and physical exercise in advanced cancer patients: A randomized controlled intervention trial. Clin Nutr. 2020 Dec;39(12):3637-3644. doi: 10.1016/j.clnu.2020.04.008. 11. Winters-Stone KM, Lyons KS, Dobek J, Dieckmann NF, Bennett JA, Nail L, Beer TM. Benefits of partnered strength training for prostate cancer survivors and spouses: results from a randomized controlled trial of the Exercising Together project. J Cancer Surviv. 2016 Aug; 10(4):633-44. doi: 10.1007/s11764-015-0509-0. 12. Gaskin CJ, Fraser SF, Owen PJ, Craike M, Orellana L, Livingston PM. Fitness outcomes from a randomised controlled trial of exercise training for men with prostate cancer: the ENGAGE study. J Cancer Surviv. 2016 Dec; 10(6):972-980. doi: 10.1007/s11764-016-0543-6. 13. Roanne J. Segal et al., Randomized Controlled Trial of Resistance or Aerobic Exercise in Men Receiving Radiation Therapy for Prostate Cancer. JCO 27, 344-351(2009). doi:10.1200/JCO.2007.15.4963. 14. Bjerre ED, Brasso K, Jørgensen AB, Petersen TH, Eriksen AR, Tolver A, Christensen JF, Poulsen MH, Madsen SS, Østergren PB, Borre M, Krustrup P, Johansen C, Rørth M, Midtgaard J. Football Compared with Usual Care in Men with Prostate Cancer (FC Prostate Community Trial): A Pragmatic Multicentre Randomized Controlled Trial. Sports Med. 2019 Jan; 49(1):145-158. doi: 10.1007/s40279-018-1031-0. |
|  |
| **Intervention** |
| 1. Alt CA, Gore EM, Montagnini ML, Ng AV. Muscle endurance, cancer-related fatigue, and radiotherapy in prostate cancer survivors. Muscle Nerve. 2011 Mar;43(3):415-24. doi: 10.1002/mus.21913. 2. Bergin ART, Hovey E, Lloyd A, Marx G, Parente P, Rapke T, de Souza P. Docetaxel-related fatigue in men with metastatic prostate cancer: a descriptive analysis. Support Care Cancer. 2017 Sep;25(9):2871-2879. doi: 10.1007/s00520-017-3706-8. 3. Cheung AS, Gray HA, Schache AG, Hoermann R, Bicknell J, Joon DL, Zajac JD, Pandy MG, Grossmann M. Biomechanical Leg Muscle Function During Stair Ambulation in Men Receiving Androgen Deprivation Therapy. J Gerontol A Biol Sci Med Sci. 2020 Sep 16;75(9):1715-1722. doi: 10.1093/gerona/glz169. 4. Cushen SJ, Power DG, Murphy KP, McDermott R, Griffin BT, Lim M, Daly L, MacEneaney P, O' Sullivan K, Prado CM, Ryan AM. Impact of body composition parameters on clinical outcomes in patients with metastatic castrate-resistant prostate cancer treated with docetaxel. Clin Nutr ESPEN. 2016 Jun;13:e39-e45. doi: 10.1016/j.clnesp.2016.04.001. 5. Kimura Y, Yamada M, Ohji S, Ishiyama D, Nishio N, Otobe Y, Koyama S, Suzuki M, Ichikawa T, Ito D, Maehori N, Nagae H. Presence of sarcopenic obesity and evaluation of the associated muscle quality in Japanese older men with prostate cancer undergoing androgen deprivation therapy. J Geriatr Oncol. 2019 Sep;10(5):835-838. doi: 10.1016/j.jgo.2019.03.017. 6. Navarro-Martínez R, Serrano-Carrascosa M, Buigues C, Fernández-Garrido J, Sánchez-Martínez V, Castelló-Domenech AB, García-Villodre L, Wong-Gutiérrez A, Rubio-Briones J, Cauli O. Frailty syndrome is associated with changes in peripheral inflammatory markers in prostate cancer patients undergoing androgen deprivation therapy. Urol Oncol. 2019 Dec;37(12):976-987. doi: 10.1016/j.urolonc.2019.08.005. 7. Smith MR, Saad F, Egerdie B, Sieber PR, Tammela TL, Ke C, Leder BZ, Goessl C. Sarcopenia during androgen-deprivation therapy for prostate cancer. J Clin Oncol. 2012 Sep 10;30(26):3271-6. doi: 10.1200/JCO.2011.38.8850. |
| **Comparator** |
| 1. Beydoun N, Bucci JA, Chin YS, Spry N, Newton R, Galvão DA. Prospective study of exercise intervention in prostate cancer patients on androgen deprivation therapy. J Med Imaging Radiat Oncol. 2014;58(3):369-76. doi: 10.1111/1754-9485.12115. 2. Dalla Via J, Owen PJ, Daly RM, Mundell NL, Livingston PM, Rantalainen T, Foulkes SJ, Millar JL, Murphy DG, Fraser SF. Musculoskeletal Responses to Exercise Plus Nutrition in Men with Prostate Cancer on Androgen Deprivation: A 12-Month RCT. Med Sci Sports Exerc. 2021 Oct 1;53(10):2054-2065. doi: 10.1249/MSS.0000000000002682. 3. Dawson JK, Dorff TB, Todd Schroeder E, Lane CJ, Gross ME, Dieli-Conwright CM. Impact of resistance training on body composition and metabolic syndrome variables during androgen deprivation therapy for prostate cancer: a pilot randomized controlled trial. BMC Cancer. 2018 Apr 3;18(1):368. doi: 10.1186/s12885-018-4306-9. 4. Galvão DA, Spry N, Denham J, Taaffe DR, Cormie P, Joseph D, Lamb DS, Chambers SK, Newton RU. A multicentre year-long randomised controlled trial of exercise training targeting physical functioning in men with prostate cancer previously treated with androgen suppression and radiation from TROG 03.04 RADAR. Eur Urol. 2014 May;65(5):856-64. doi: 10.1016/j.eururo.2013.09.041. 5. Inglis JE, Fernandez ID, van Wijngaarden E, Culakova E, Reschke JE, Kleckner AS, Lin PJ, Mustian KM, Peppone LJ. Effects of High-Dose Vitamin D Supplementation on Phase Angle and Physical Function in Patients with Prostate Cancer on ADT. Nutr Cancer. 2021;73(10):1882-1889. doi: 10.1080/01635581.2020.1819348. 6. Kim SH, Seong DH, Yoon SM, Choi YD, Choi E, Song Y, Song H. The Effect on Bone Outcomes of Home-based Exercise Intervention for Prostate Cancer Survivors Receiving Androgen Deprivation Therapy: A Pilot Randomized Controlled Trial. Cancer Nurs. 2018 Sep/Oct;41(5):379-388. doi: 10.1097/NCC.0000000000000530. 7. Lopez P, Taaffe DR, Newton RU, Spry N, Joseph D, Tang C, Buffart LM, Galvão DA. Reporting Attendance and Resistance Exercise Compliance in Men with Localized Prostate Cancer. Med Sci Sports Exerc. 2023 Mar 1;55(3):354-364. doi: 10.1249/MSS.0000000000003069. 8. Newton RU, Galvão DA, Spry N, Joseph D, Chambers SK, Gardiner RA, Hayne D, Taaffe DR. Timing of exercise for muscle strength and physical function in men initiating ADT for prostate cancer. Prostate Cancer Prostatic Dis. 2020 Sep;23(3):457-464. doi: 10.1038/s41391-019-0200-z. 9. Park YH, Lee JI, Lee JY, Cheong IY, Hwang JH, Seo SI, Lee KH, Yoo JS, Chung SH, So Y. Internet of things-based lifestyle intervention for prostate cancer patients on androgen deprivation therapy: a prospective, multicenter, randomized trial. Am J Cancer Res. 2021 Nov 15;11(11):5496-5507. 10. Schumacher O, Galvão DA, Taaffe DR, Spry N, Hayne D, Tang C, Chee R, Newton RU. Nationwide Industry-Led Community Exercise Program for Men With Locally Advanced, Relapsed, or Metastatic Prostate Cancer on Androgen-Deprivation Therapy. JCO Oncol Pract. 2022 Aug;18(8):e1334-e1341. doi: 10.1200/OP.21.00745. Epub 2022 May 18. 11. Smith MR, Goode M, Zietman AL, McGovern FJ, Lee H, Finkelstein JS. Bicalutamide monotherapy versus leuprolide monotherapy for prostate cancer: effects on bone mineral density and body composition. J Clin Oncol. 2004 Jul 1;22(13):2546-53. doi: 10.1200/JCO.2004.01.174. 12. Taaffe DR, Buffart LM, Newton RU, Spry N, Denham J, Joseph D, Lamb D, Chambers SK, Galvão DA. Time on androgen deprivation therapy and adaptations to exercise: secondary analysis from a 12-month randomized controlled trial in men with prostate cancer. BJU Int. 2018 Feb;121(2):194-202. doi: 10.1111/bju.14008. 13. Taaffe DR, Galvão DA, Spry N, Joseph D, Chambers SK, Gardiner RA, Hayne D, Cormie P, Shum DHK, Newton RU. Immediate versus delayed exercise in men initiating androgen deprivation: effects on bone density and soft tissue composition. BJU Int. 2019 Feb;123(2):261-269. doi: 10.1111/bju.14505. 14. Wang C, Cunningham G, Dobs A, Iranmanesh A, Matsumoto AM, Snyder PJ, Weber T, Berman N, Hull L, Swerdloff RS. Long-term testosterone gel (AndroGel) treatment maintains beneficial effects on sexual function and mood, lean and fat mass, and bone mineral density in hypogonadal men. J Clin Endocrinol Metab. 2004 May;89(5):2085-98. doi: 10.1210/jc.2003-032006. 15. Wilson RL, Taaffe DR, Newton RU, Hart NH, Lyons-Wall P, Galvão DA. Maintaining Weight Loss in Obese Men with Prostate Cancer Following a Supervised Exercise and Nutrition Program-A Pilot Study. Cancers (Basel). 2021 Jul 7;13(14):3411. doi: 10.3390/cancers13143411. 16. Winters-Stone KM, Dobek JC, Bennett JA, Dieckmann NF, Maddalozzo GF, Ryan CW, Beer TM. Resistance training reduces disability in prostate cancer survivors on androgen deprivation therapy: evidence from a randomized controlled trial. Arch Phys Med Rehabil. 2015 Jan;96(1):7-14. doi: 10.1016/j.apmr.2014.08.010. 17. Lim JJ et al., Lifestyle Intervention for promoting physical activity in prostate cancer patients with androgen deprivation therapy. Journal of Men's Health. 2020. 16(4);54-64. |
| **Outcome** |
| 1. Nilsen TS, Thorsen L, Kirkegaard C, Ugelstad I, Fosså SD, Raastad T. The effect of strength training on muscle cellular stress in prostate cancer patients on ADT. Endocr Connect. 2016 Mar;5(2):74-82. doi: 10.1530/EC-15-0120. 2. Nobes JP, Langley SE, Klopper T, Russell-Jones D, Laing RW. A prospective, randomized pilot study evaluating the effects of metformin and lifestyle intervention on patients with prostate cancer receiving androgen deprivation therapy. BJU Int. 2012 May;109(10):1495-502. doi: 10.1111/j.1464-410X.2011.10555.x. 3. Uth J, Hornstrup T, Christensen JF, Christensen KB, Jørgensen NR, Helge EW, Schmidt JF, Brasso K, Helge JW, Jakobsen MD, Andersen LL, Rørth M, Midtgaard J, Krustrup P. Football training in men with prostate cancer undergoing androgen deprivation therapy: activity profile and short-term skeletal and postural balance adaptations. Eur J Appl Physiol. 2016 Mar;116(3):471-80. doi: 10.1007/s00421-015-3301-y. 4. Wibowo E, Wassersug RJ, Robinson JW, Matthew A, McLeod D, Walker LM. How Are Patients With Prostate Cancer Managing Androgen Deprivation Therapy Side Effects? Clin Genitourin Cancer. 2019 Jun;17(3):e408-e419. doi: 10.1016/j.clgc.2018.12.006. Epub 2018 Dec 20. |
| **Study Design** |
| 1. Bergerot CD et al., The effects of bright white light therapy on obese frailty in older men with prostate cancer on hormonal therapy: A pilot randomized control trial. JCO. 39, 75-75(2021). doi:10.1200/JCO.2021.39.6_suppl.75. 2. Cavka L, Pohar Perme M, Zakotnik B, Rotovnik Kozjek N, Seruga B. Nutritional Status and Health-Related Quality of Life in Men with Advanced Castrate-Resistant Prostate Cancer. Nutr Cancer. 2022;74(2):472-481. doi: 10.1080/01635581.2021.1884731. 3. Edelman MJ, Gandara DR, Meyers FJ, Ishii R, O'Mahony M, Uhrich M, Lauder I, Houston J, Gietzen DW. Serotonergic blockade in the treatment of the cancer anorexia-cachexia syndrome. Cancer. 1999 Aug 15;86(4):684-8. 4. Edmunds K, Reeves P, Scuffham P, Galvão DA, Newton RU, Jones M, Spry N, Taaffe DR, Joseph D, Chambers SK, Tuffaha H. Cost-Effectiveness Analysis of Supervised Exercise Training in Men with Prostate Cancer Previously Treated with Radiation Therapy and Androgen-Deprivation Therapy. Appl Health Econ Health Policy. 2020 Oct;18(5):727-737. doi: 10.1007/s40258-020-00564-x. 5. Fairman CM, Kendall KL, Newton RU, Hart NH, Taaffe DR, Chee R, Tang CI, Galvão DA. Examining the effects of creatine supplementation in augmenting adaptations to resistance training in patients with prostate cancer undergoing androgen deprivation therapy: a randomised, double-blind, placebo-controlled trial. BMJ Open. 2019 Sep 20;9(9):e030080. doi: 10.1136/bmjopen-2019-030080. 6. Galvão DA, Nosaka K, Taaffe DR, Spry N, Kristjanson LJ, McGuigan MR, Suzuki K, Yamaya K, Newton RU. Resistance training and reduction of treatment side effects in prostate cancer patients. Med Sci Sports Exerc. 2006 Dec;38(12):2045-52. doi: 10.1249/01.mss.0000233803.48691.8b. 7. Galvão DA, Spry N, Taaffe DR, Denham J, Joseph D, Lamb DS, Levin G, Duchesne G, Newton RU. A randomized controlled trial of an exercise intervention targeting cardiovascular and metabolic risk factors for prostate cancer patients from the RADAR trial. BMC Cancer. 2009 Dec 2; 9:419. doi: 10.1186/1471-2407-9-419. 8. Galvão DA, Taaffe DR, Spry N, Joseph D, Newton RU. Acute versus chronic exposure to androgen suppression for prostate cancer: impact on the exercise response. J Urol. 2011 Oct; 186(4):1291-7. doi: 10.1016/j.juro.2011.05.055. Epub 2011 Aug 17. 9. Hanson ED, Sheaff AK, Sood S, Ma L, Francis JD, Goldberg AP, Hurley BF. Strength training induces muscle hypertrophy and functional gains in black prostate cancer patients despite androgen deprivation therapy. J Gerontol A Biol Sci Med Sci. 2013 Apr; 68(4):490-8. doi: 10.1093/gerona/gls206. Epub 2012 Oct 22. 10. Harrison ED, et al. EXTEND: Safety and efficacy of exercise training in men receiving enzalutamide (ENZ) in combination with conventional androgen deprivation therapy (ADT) for hormone na+»ve prostate cancer (HSPC). Annals of Oncology. 2018; 29 (suppl. 8). doi: 10.1093/annonc/mdy300.064. 11. Kiwata JL, Dorff TB, Todd Schroeder E, Salem GJ, Lane CJ, Rice JC, Gross ME, Dieli-Conwright CM. A pilot randomised controlled trial of a periodised resistance training and protein supplementation intervention in prostate cancer survivors on androgen deprivation therapy. BMJ Open. 2017 Jul 10;7(7):e016910. doi: 10.1136/bmjopen-2017-016910. 12. Macciò A, Gramignano G, Madeddu C. A Multitargeted Treatment Approach for Anemia and Cachexia in Metastatic Castration-Resistant Prostate Cancer. J Pain Symptom Manage. 2015 Aug;50(2):e1-4. doi: 10.1016/j.jpainsymman.2015.04.014. 13. Newton RU, Jeffery E, Galvão DA, Peddle-McIntyre CJ, Spry N, Joseph D, Denham JW, Taaffe DR. Body composition, fatigue and exercise in patients with prostate cancer undergoing androgen-deprivation therapy. BJU Int. 2018 Dec;122(6):986-993. doi: 10.1111/bju.14384. 14. Newton RU, Taaffe DR, Spry N, Gardiner RA, Levin G, Wall B, Joseph D, Chambers SK, Galvão DA. A phase III clinical trial of exercise modalities on treatment side-effects in men receiving therapy for prostate cancer. BMC Cancer. 2009 Jun 29;9:210. doi: 10.1186/1471-2407-9-210. 15. Nilsen TS, Johansen SH, Thorsen L, Fairman CM, Wisløff T, Raastad T. Does Androgen Deprivation for Prostate Cancer Affect Normal Adaptation to Resistance Exercise? Int J Environ Res Public Health. 2022 Mar 23;19(7):3820. doi: 10.3390/ijerph19073820. 16. Owen PJ, Daly RM, Livingston PM, Mundell NL, Dalla Via J, Millar JL, Fraser SF. Efficacy of a multi-component exercise programme and nutritional supplementation on musculoskeletal health in men treated with androgen deprivation therapy for prostate cancer (IMPACT): study protocol of a randomised controlled trial. Trials. 2017 Oct 3;18(1):451. doi: 10.1186/s13063-017-2185-z. 17. Palumbo C, Antonelli A, Triggiani L, Dalla Volta A, Maffezzoni F, Zamboni S, Borghetti P, Rinaudo L, Valcamonico F, Maroldi R, Magrini SM, Simeone C, Berruti A; Collaborators. Changes in body composition and lipid profile in prostate cancer patients without bone metastases given Degarelix treatment: the BLADE prospective cohort study. Prostate Cancer Prostatic Dis. 2021 Sep;24(3):852-859. doi: 10.1038/s41391-021-00345-0. 18. Skinner TL, Peeters GG, Croci I, Bell KR, Burton NW, Chambers SK, Bolam KA. Impact of a brief exercise program on the physical and psychosocial health of prostate cancer survivors: A pilot study. Asia Pac J Clin Oncol. 2016 Sep;12(3):225-34. doi: 10.1111/ajco.12474. 19. Taaffe DR, Newton RU, Spry N, Joseph DJ, Galvão DA. Responsiveness to Resistance-Based Multimodal Exercise Among Men With Prostate Cancer Receiving Androgen Deprivation Therapy. J Natl Compr Canc Netw. 2019;17(10):1211-1220. doi:10.6004/jnccn.2019.7311. 20. Tassinari D, Fochessati F, Panzini I, Poggi B, Sartori S, Ravaioli A. Rapid progression of advanced "hormone-resistant" prostate cancer during palliative treatment with progestins for cancer cachexia. J Pain Symptom Manage. 2003 May;25(5):481-4. doi: 10.1016/s0885-3924(03)00043-5. 21. Van Veldhuizen PJ, Taylor SA, Williamson S, Drees BM. Treatment of vitamin D deficiency in patients with metastatic prostate cancer may improve bone pain and muscle strength. J Urol. 2000 Jan;163(1):187-90. doi: 10.1097/00005392-200001000-00044. 22. Wadhwa VK, Weston R, Parr NJ. Bicalutamide monotherapy preserves bone mineral density, muscle strength and has significant health-related quality of life benefits for osteoporotic men with prostate cancer. BJU Int. 2011 Jun;107(12):1923-9. doi: 10.1111/j.1464-410X.2010.09726.x. 23. Wilson RL, Newton RU, Taaffe DR, Hart NH, Lyons-Wall P, Galvão DA. Weight Loss for Obese Prostate Cancer Patients on Androgen Deprivation Therapy. Med Sci Sports Exerc. 2021 Mar 1;53(3):470-478. doi: 10.1249/MSS.0000000000002509. 24. Winters-Stone KM, Lyons KS, Nail LM, Beer TM. The Exercising Together project: design and recruitment for a randomized, controlled trial to determine the benefits of partnered strength training for couples coping with prostate cancer. Contemp Clin Trials. 2012 Mar;33(2):342-50. doi: 10.1016/j.cct.2011.10.015. Epub 2011 Nov 11. |
