# Supplementary material for: Effectiveness and Safety of Interventions for Sarcopenia in Advanced Prostate Carcinoma: Systematic Review
Source: J Cachexia Sarcopenia Muscle. 2026 May 5;17(3):e70290. doi: 10.1002/jcsm.70290 (PMC13144553; doi:10.1002/jcsm.70290)
Supplement: Supplementary file 5 — Table S3: Results of all meta‐analysis and subgroup analysis of exercise training (ET) interventions and analysis not reported in the manuscript regarding other interventions [file JCSM-17-e70290-s004.docx]

Supplementary Table S3. Results of all meta-analysis and subgroup analysis of exercise training (ET) interventions and analysis not reported in the manuscript regarding other interventions

| **Outcome measure** | **Model** | **K** |  | **MD** | **95% CI** | **I^2^ (%)** | **Test for subgroup differences (%)**  **(P-value)** |
| --- | --- | --- | --- | --- | --- | --- | --- |
| **Survival** |  |  |  |  |  |  |  |
| ***Cancer-specific survival (Hazard Ratio)*** | | | | | | | |
| **Low-dose Megesterol Acetate** |  | 1 |  | 0.962 | 0.531, 1,743 | NA |  |
| **High-dose Megesterol Acetate** |  | 1 |  | 1.108 | 0.606, 2.008 | NA |  |
| **Muscle Mass** |  |  |  |  |  |  |  |
| ***Lean body mass (kg)*** | | | | | | | |
| **Exercise Training** | Random | 12 |  | -1.29 | -4.06, 1.48 | 71 |  |
| ***Sensitivity analysis*** |  |  |  |  |  |  |  |
| *Removing Sajid 2016* | Fixed | 10 |  | -0.06 | -1.21, 1.09 | 25 |  |
| ***Subgroup analysis*** |  |  |  |  |  |  | 10.16 (<0.01) |
| Resistance | Fixed | 3 |  | 3.22 | 0.69, 5.75 | 0 |  |
| Resistance and aerobic | Fixed | 6 |  | -0.44 | -1.88, 1.00 | 0 |  |
| Football training | Fixed | 1 |  | -2.80 | -5.69, 0.09 | NA |  |
| **Lower muscle strength** | | | | | | | |
| ***Knee extension (1-RM, kg)*** | | | | | | | |
| **Exercise Training** | Fixed | 3 |  | 3.41 | -3.55, 10.37 | 26 |  |
| ***Leg extension (1-RM, kg)*** | | | | | | | |
| **Exercise Training** | Fixed | 3 |  | 9.63 | 4.83, 14.42 | 0 |  |
| **ET+PRO (% kg)** | Fixed | 1 |  | 21 | 10.65, 31.35 | NA |  |
| ***Leg press (1-RM, kg)*** | | | | | | | |
| **Exercise Training** | Random | 5 |  | 25.17 | 8.71, 41.62 | 51 |  |
| **ET+PRO (% kg)** | Fixed | 1 |  | 18 | 11.1, 24.9 | NA |  |
| **Upper muscle strength** | | | | | | | |
| ***Chest press (1-RM, kg)*** | | | | | | | |
| **Exercise Training** | Fixed | 6 |  | 1.75 | -1.40, 4.90 | 0 |  |
| ***Sensitivity analysis*** | | | | | | | |
| *Removing Sajid 2016* | Fixed | 4 |  | 1.70 | -1.48, 4.88 | 0 |  |
| ***Seated row (1-RM, kg)*** | | | | | | | |
| **Exercise Training** | Fixed | 4 |  | 4.38 | 1.54, 7.22 | 5 |  |
| ***Shoulder press (1-RM, kg)*** | | | | | | | |
| **Exercise Training** | Fixed | 1 |  | 5.00 | -0.47, 10,47 | NA |  |
| ***Handgrip strength (1-RM, kg)*** | | | | | | | |
| **Exercise Training** | Random | 3 |  | 0.34 | -5.66, 6.34 | 68 |  |
| **Physical functioning** | | | | | | | |
| ***400m walk (s)*** | | | | | | | |
| **Exercise Training** | Fixed | 5 |  | -7.48 | -17.49, 2.54 | 0 |  |
| ***6m walk at usual pace (s)*** | | | | | | | |
| **Exercise Training** | Random | 3 |  | -0.03 | -0.35, 0.29 | 60 |  |
| ***6m walk at fast pace (s)*** | | | | | | | |
| **Exercise Training** | Fixed | 3 |  | -0.04 | -0.19, 0.12 | 19 |  |
| ***Steps per day (n steps)*** | | | | | | | |
| **Exercise Training** | Random | 2 |  | 3348.64 | -1510.27, 8207.55 | 55 |  |
| ***Chair sit-to-stand test, 5 repetitions (s)*** | | | | | | | |
| **Exercise Training** | Fixed | 4 |  | -1.02 | -1.70, -0.34 | 0 |  |
| ***Chair sit-to-stand test, 30 seconds (n repetitions)*** | | | | | | | |
| **Exercise Training** | Fixed | 3 |  | 1.43 | 0.22, 2.65 | 0 |  |
| **ET + PRO** | Fixed | 1 |  | -0.80 | -2.93, 1.33 | NA |  |
| ***Timed up-and-go test (s)*** | | | | | | | |
| **Exercise Training** | Fixed | 5 |  | 0.50 | -0.17, 1.16 | 0 |  |
| **ET + PRO** | Fixed | 1 |  | 0.50 | -0.37, 1.37 | NA |  |
| ***Stair climb (s)*** | | | | | | | |
| **Exercise Training** | Fixed | 5 |  | -0.12 | -0.37, 0.12 | 0 |  |
| **ET+PRO** | Fixed | 1 |  | 0.80 | -1.02, 2.62 | NA |  |
| ***Jump height (cm)*** | | | | | | | |
| **Exercise Training** | Fixed | 1 |  | 1.00 | -1.53, 3.53 | NA |  |
| ***SPPB score*** | | | | | | | |
| **Exercise Training** | Fixed | 2 |  | 3.04 | -0.38, 6.45 | 0 |  |
| **Balance** | | | | | | | |
| ***6m backwards walk (s)*** | | | | | | | |
| **Exercise Training** | Fixed | 4 |  | -1.25 | -3.89, 1.38 | 0 |  |
| ***SOT score*** | | | | | | | |
| **Exercise Training** | Fixed | 3 |  | -0.84 | -4.07, 2.38 | 0 |  |
| ***ABC score*** | | | | | | | |
| **Exercise Training** | Fixed | 2 |  | -0.89 | -11.04, 9.26 | 0 |  |
| ***Flamingo test (s)*** | | | | | | | |
| **Exercise Training** | Fixed | 1 |  | -1.00 | -6.59, 4.59 | NA |  |
| ***Bipedal stance sway area (mm^2^)*** | | | | | | | |
| **Exercise Training** | Fixed | 1 |  | 83.80 | -271.65, 439.25 | NA |  |
| ***Tandem stance sway area (mm^2^)*** | | | | | | | |
| **Exercise Training** | Fixed | 1 |  | -155.20 | -675.89, 365.49 | NA |  |
| **HRQOL** | | | | | | | |
| ***SF-36 Physical health composite score*** | | | | | | | |
| **Exercise Training** | Fixed | 2 |  | -1.28 | -4.62, 2.05 | 0% |  |
| ***SF-36 Mental health composite score*** | | | | | | | |
| **Exercise Training** | Fixed | 2 |  | 3.52 | -0.25, 7.28 | 0% |  |
| ***SF-36 Physical functioning domain*** | | | | | | | |
| **Exercise Training** | Random | 2 |  | 1.96 | -3.33, 7.25 | 78% |  |
| ***QLQ-C30 global score*** | | | | | | | |
| **Exercise Training** | Fixed | 2 |  | 0.54 | -6.47, 7.55 | 0% |  |
| ***QLQ-C30 cognitive domain score*** | | | | | | | |
| **Exercise Training** | Fixed | 2 |  | 7.78 | 1.08, 14.47 | 29% |  |
| ***QLQ-C30 emotional domain score*** | | | | | | | |
| **Exercise Training** | Fixed | 2 |  | 9.25 | 3.02, 15.49 | 0% |  |
| ***QLQ-C30 physical domain score*** | | | | | | | |
| **Exercise Training** | Fixed | 2 |  | 5.65 | 0.02, 11.28 | 0% |  |
| ***QLQ-C30: Appetite loss score*** | | | | | | | |
| **Exercise Training** | Fixed | 2 |  | 0.30 | -5.84, 6.44 | 0% |  |
| ***QLQ-C30: Constipation score*** | | | | | | | |
| **Exercise Training** | Fixed | 2 |  | 0.37 | -7.19, 7.93 | 0% |  |
| ***QLQ-C30: Diarrhea score*** | | | | | | | |
| **Exercise Training** | Fixed | 2 |  | 3.97 | -6.82, 14.75 | 56% |  |
| ***QLQ-C30: Dyspnea score*** | | | | | | | |
| **Exercise Training** | Fixed | 2 |  | -12.58 | -22.10, -3.06 | 0% |  |
| ***QLQ-C30: Insomnia score*** | | | | | | | |
| **Exercise Training** | Fixed | 2 |  | -4.75 | -15.64, 6.13 | 0% |  |
| ***QLQ-C30: Pain score*** | | | | | | | |
| **Exercise Training** | Fixed | 2 |  | 0.81 | -7.63, 9.25 | 0% |  |
| ***QLQ-C30: Role score*** | | | | | | | |
| **Exercise Training** | Fixed | 2 |  | 5.69 | -1.80, 13.17 | 0% |  |
| ***QLQ-C30: Social score*** | | | | | | | |
| **Exercise Training** | Fixed | 2 |  | 4.10 | -4.39, 12.58 | 0% |  |
| ***QLQ-C30: Fatigue score*** | | | | | | | |
| **Exercise Training** | Random | 2 |  | -4.86 | -16.68, 6.96 | 57% |  |
| ***QLQ-C30: Nausea score*** | | | | | | | |
| **Exercise Training** | Random | 2 |  | -1.90 | -8.55, 4.75 | 67% |  |
| ***QLQ-PR25: Bowel symptoms score*** | | | | | | | |
| **Exercise Training** | Fixed | 2 |  | -1.64 | -4.02, 0.73 | 0% |  |
| ***QLQ-PR25: Sexual activity score*** | | | | | | | |
| **Exercise Training** | Fixed | 2 |  | 5.40 | -1.94, 12.74 | 0% |  |
| ***QLQ-PR25: Sexual function score*** | | | | | | | |
| **Exercise Training** | Fixed | 2 |  | 16.92 | 10.41, 23.44 | 0% |  |
| ***QLQ-PR25: Treatment score*** | | | | | | | |
| **Exercise Training** | Fixed | 2 |  | -4.52 | -8.99, -0.05 | 0% |  |
| ***QLQ-PR25: Urinary symptoms score*** | | | | | | | |
| **Exercise Training** | Random | 2 |  | -8.79 | -24.05, 6.46 | 0% |  |
| ***FACT-G score*** | | | | | | | |
| **Exercise Training** | Fixed | 1 |  | 17.90 | 15.11, 20.69 | NA |  |
| **Lifestyle Intervention** | Fixed | 1 |  | 5.00 | -3.45, 13.45 | NA |  |
| ***FACT-P score*** | | | | | | | |
| **Exercise Training** | Fixed | 2 |  | 2.20 | -2.26, 6.67 | 0% |  |
| **Lifestyle Intervention** | Fixed | 1 |  | 7.00 | -5.04, 19.04 | NA |  |
| **Fatigue** | | | | | | | |
| ***FACT-F score*** | | | | | | | |
| **Exercise Training** | Random | 4 |  | 6.70 | -1.86, 15.25 | 97% |  |
| ***Sensitivity analysis*** |  |  |  |  |  |  |  |
| *Removing Hojan, 2016* | Random | 3 |  | 1.92 | -0.40, 4.23 | 0% |  |
| ***MSFI-SF score*** | | | | | | | |
| **Exercise Training** | Fixed | 1 |  | 5.00 | -12.61, 22.61 | NA |  |
| **Psychological Distress** | | | | | | | |
| ***BSI-18: Global Severity Index score*** | | | | | | | |
| **Exercise Training** | Fixed | 4 |  | -1.63 | -3.10, -0.15 | 0% |  |
| ***BSI-18: Anxiety score*** | | | | | | | |
| **Exercise Training** | Fixed | 4 |  | -0.49 | -1.05, 0.08 | 0% |  |
| ***BSI-18: Depression score*** | | | | | | | |
| **Exercise Training** | Fixed | 4 |  | -0.39 | -1.06, 0.28 | 0% |  |
| ***BSI-18: Somatization score*** | | | | | | | |
| **Exercise training** | Fixed | 4 |  | -0.69 | -1.32, -0.07 | 0% |  |

1-RM: one repetition maximum weight lifted; Note: Fixed: Fixed effects model; MD: mean difference; NA: Not applicable; Random: Random effects model
